# Proteomic profiling of the rat hippocampus from the kindling and pilocarpine models of epilepsy: potential targets in calcium regulatory network

**DOI:** 10.1038/s41598-021-87555-7

**Published:** 2021-04-15

**Authors:** Leila Sadeghi, Albert Anatolyevich Rizvanov, Bahareh Dabirmanesh, Ilnur Ildusovich Salafutdinov, Mohammad Sayyah, Amir Shojaei, Javad Zahiri, Javad Mirnajafi-Zadeh, Babak Khorsand, Khosro Khajeh, Yaghoub Fathollahi

**Affiliations:** 1 Department of Biochemistry, Faculty of Biological Science, Tarbiat Modares University, Tehran, Iran; 2 Department of Animal Biology, Faculty of Natural Science, University of Tabriz, Tabriz, Iran; 3 Institute of Fundamental Medicine and Biology, Kazan Federal University, Kazan, Russian Federation; 4 Department of Physiology and Pharmacology, Pasteur Institute of Iran, Tehran, Iran; 5 Department of Medical Physiology, Faculty of Medical Science, Tarbiat Modares University, Tehran, Iran; 6 Department of Biophysics, Faculty of Biological Science, Tarbiat Modares University, Tehran, Iran; 7 Department of Computer Engineering, Faculty of Engineering, Ferdowsi University of Mashhad, Mashhad, Iran

**Keywords:** Neurochemistry, Diseases of the nervous system

## Abstract

Herein proteomic profiling of the rat hippocampus from the kindling and pilocarpine models of epilepsy was performed to achieve new potential targets for treating epileptic seizures. A total of 144 differently expressed proteins in both left and right hippocampi by two-dimensional electrophoresis coupled to matrix-assisted laser desorption-mass spectrometry were identified across the rat models of epilepsy. Based on network analysis, the majority of differentially expressed proteins were associated with Ca^2+^ homeostasis. Changes in ADP-ribosyl cyclase (ADPRC), lysophosphatidic acid receptor 3 (LPAR3), calreticulin, ubiquitin carboxyl-terminal hydrolase L1 (UCH-L1), synaptosomal nerve-associated protein 25 (SNAP 25) and transgelin 3 proteins were probed by Western blot analysis and validated using immunohistochemistry. Inhibition of calcium influx by 8-Bromo-cADP-Ribose (8-Br-cADPR) and 2-Aminoethyl diphenylborinate (2-APB) which act via the ADPRC and LPAR3, respectively, attenuated epileptic seizures. Considering a wide range of molecular events and effective role of calcium homeostasis in epilepsy, polypharmacy with multiple realistic targets should be further explored to reach the most effective treatments.

## Introduction

Epilepsy is a disorder of the brain with lasting susceptibility to generate epileptic seizures that affects millions of peoples all over the world^1^. Temporal lobe epilepsy (TLE) is one of the common type of epilepsy that is known by severe hippocampal sclerosis. Current treatments of epilepsy alleviant symptoms and are unable to cure epilepsy^2^, because yet its exact mechanism is still unknown^3, 4^. Considerable research on epilepsy has therefore been oriented to dissect out the molecular mechanisms underlying TLE^4^. For this purpose, the use of convenient animal models is needed. The kindling (chemical or electrical) and the pilocarpine models are the most frequently used animal models of temporal lobe epilepsic seizures or chronic epilepsy^5, 6^. The pilocarpine model of TLE replicates general progressive of events as observed in human TLE^7^. Kindling is caused by repetitive administration of convulsant agents (pentylenetetrazol or electrical stimuli) to reduce seizure threshold in animals, similar to the process that take place in human TLE^5^. These models may provide useful insights into the activity of epileptic circuits and future treatment strategies for epilepsy^5, 6^.

To achieve an effective treatment, molecular level approaches can be used to identify changes in gene or protein expression levels underlying epileptic state, which provides new perspectives for the discovery of new anti-seizure drugs^8^. A wealth of experimental evidence has indicated that the hippocampus is the main brain region for generation, propagation and termination of epileptic seizures^3^. So proteomic profiling of the rat hippocampus from the kindling and pilocarpine models of epilepsy using 2-dimensional gel electrophoresis (2-DE) and matrix-assisted laser desorption-mass spectrometry (MALDI-TOF/TOF) was performed and the protein expression network was analyzed using systems biology approaches.

An excessive increase in Ca^2+^ content of the epileptic brain from various resources can trigger some pathophysiological aspects of epileptic seizure^9, 10^. Proteomics data analysis and systems biology results revealed an important role played by calcium influx in cells damage processes^11^. A decrease in the UCH-L1 expression level and an increase in cytosolic free Ca^2+^, may be the cause of a higher sensitivity of the left hippocampus to seizure and its lower seizure threshold as compared to the right hippocampus^12^. In the treatment of epilepsy, the role played by ADP-ribosylcyclase (ADPRC) and lysophosphatidic acid receptor 3 (LPAR3) as components of Ca^2+^ influx pathways has yet not been demonstrated. Therefore, changes in ADPRC and LPAR3 proteins across the 3 rat models of epilepsy were probed by Western blot analysis and validated using immunohistochemistry. 8-Bromo-cADP-Ribose (8-Br-cADPR) and 2-Aminoethyl diphenylborinate (2-APB) were used to assess epileptic seizures via inhibiting ADPRC and LPAR3 pathways, respectively.

## Material and methods

### Animal models

In vivo study was performed on adult male Wistar rats (Pasture Institute, Tehran, Iran) weighing 250–300 g (3–5 months old) kept at 20–25 °C with natural light–dark cycle and free access to municipal tap water and the commercially obtained diet.

### Ethical statement

This study includes animal experiments which has been done according to institutional and national guidelines for the care and use of laboratory animals and also ARRIVE guidelines. We tried to use minimum number of animals in this study. The study was approved by the Ethics Committee of Pasteur Institute (Tehran, Iran) and was reconciled to the European Communities Council Directive of 24 November 1986 (86/609/EEC).

The kindling and pilocarpine models of epilepsy were employed. Pilocarpine was administered according to Cavalheiro et al.^7^. Thirty minutes prior to pilocarpine administration, rats (n = 24) were received a subcutaneous methyl scopolamine (1 mg/kg) to reduce peripheral cholinergic effects of pilocarpine. Then a single dose of pilocarpine (380 mg/kg in 0.1 ml saline, i.p.) was injected and pilocarpine-induced seizures were assessed using modified Racine’s scale^13^. A rat experiencing continuous seizure activity during 2 h after pilocarpine injection was considered as a case of status epilepticus (SE). The SE phase was initiated about 30 min after pilocarpine injection. SE (acute phase) was stopped after two hours ongoing seizure by 10 mg/kg diazepam injection. Rats were observed for 4 h in each day after pilocarpine injection for spontaneous seizure appearance indicating the occurrence of the chronic phase of SE^14^. Based on the previous scale, spontaneous seizures were equivalent to stage 3–5. Chronic phase started 22 ± 5 days after acute phase. Behavioral seizures were monitored for at least 30 days after acute phase. Hippocampus samples were separated four hours after the animals reached stage 4 or stage 5 behavioral seizures.

The PTZ kindling was performed according to methods previously described^15^. Animals (n = 13) were kindled by intraperitoneal injection of PTZ (35 mg/kg; 0.1 ml/100 g body weight) every day for a number of days. Following each PTZ injection, animal behaviors were observed for 30 min. The seizure stages were evaluated according to the Racine’s scale^13^. The animals that revealed three successive stage 4 or 5 seizures were considered fully kindled. The whole hippocampus were removed from the full kindled animals four hours after the occurrence of the third seizure and stored at − 80 °C freezer.

The electrical kindling was carried out according to the method of Sayyah et al.^16^. Rats (n = 15) were anesthetized with ketamine (60 mg/kg; Rotex Medica, Germany) and xylazine (10 mg/kg; Chanelle, Ireland). Then, they were implanted stereotaxically with bipolar exciting and monopolar recording electrodes (Teflon-coated stainless-steel wire twisted into a tripolar configuration) in left hemisphere of basolateral amygdala (A, − 2.5 mm from bregma; L, 4.8 mm from bregma and V, 7.5 mm from dura). Electrodes were fixed to the skull using dental acrylic. After 10 days recovery, after-discharge (AD) threshold was determined in the amygdala by a 2-s, 100-Hz monophasic square-wave stimulus of 1 ms per wave. The stimulation was initially delivered at 50 µA and then at 5-min intervals, raised stimulus intensity in increments of 10 µA were delivered, until at least 5 s of AD was recorded^16^. The mean AD threshold obtained for the animals was in the range of 50–150 µA. Then, animals were stimulated at AD threshold once daily, until three consecutive stage 5 seizures were elicited according to the Racine’s classification that known as full kindled animals^13^. The whole hippocampus were removed from the full kindled animals with the occurrence of 3 consecutive seizures four hours after the occurrence of the third seizure and stored at − 80 °C freezer.

Control group was injected with saline instead of pilocarpine (n = 3) or PTZ (n = 3). In the case of electrical kindling, control rats were implanted by electrode without stimuli (n = 3).

Control and epileptic rats were sacrificed four hours after last seizure using ketamine/xylazine anesthesia. The brains were removed from the skull and the whole hippocampus were dissected out and stored at − 80 °C freezer.

### Sample preparation

Hippocampus was homogenized with a glass/Teflon homogenizer in 0.3 mL of 20 mM Tris, 7 M urea, 2 M thiourea, 4% w/v CHAPS, 10 mM DTT, 1 mM EDTA, 1 mM PMSF and phosphatase inhibitors (0.2 mM Na_2_VO_3_ and 1 mM NaF)^17^. The suspension was sonicated for 40 s, following 1 h incubation on ice and centrifugation at 12,000*g* for 20 min. The protein concentration in the supernatant was determined using the Bradford method^18^.

### 2-DE

2-DE process was performed according to our previous experiment^17^. Briefly extracted protein samples were diluted in rehydration buffer and applied on immobilized 18 cm pH 3–10 linear gradient strips in a passive rehydration process. Previously optimized program was used in Isoelectric focusing (IEF) in order to separate proteins based on their pI. After IEF separation, the gel strips were equilibrated in a solution (containing urea, SDS, Tris, glycerol, DTT with a trace of bromophenol blue)^17^. Following the equilibration, gel strips were loaded onto the top of a 12.5% SDS polyacrylamide gel and SDS-PAGE was performed for 14 h at 20–50 mA. After the second dimension, the gels were were stained with colloidal coomassie Blue method^19^. 2-DE was performed for each sample individually as biological replicates with at least three technical replicates.

### Informatics and statistics for 2-D gel electrophoresis images analysis

Coomassie stained gels were scanned on ImageScanner III from GE Healthcare at 300 DPI resolution. The resulting TIFF images were analyzed using the ImageMaster 2D Platinum v7.0 gel analysis software-GE Healthcare Life Sciences. Protein spots presenting differences in their expression level were outlined and matched empirically (automatically and then manually), after careful examination and background subtraction. Only these selected spots were statistically evaluated using the paired t-test. The volume intensity of each spot was normalized by dividing it by the total volume intensity of spots. Total-spot volume was calculated by the software, and this referred to the sum volume of all the spots on the gel. The relative volume (%) of the variably expressed spots/proteins were figured out and the mean ratios of these spots (%) and standard deviations (SD) were used to elucidate the expression changes. Significant spots that were changed consistently and at least 2.0 fold difference in the relative volume (%) between the groups and two laterals were selected for protein identification.

### In-gel protein digestion and MALDI–TOF–TOF/MS analysis

In-gel digestion was done by Promega trypsin according to our previous work^17^. Briefly, the selected protein spots from coomassie-stained 2-D gels were excised and destained with 50 mM ammonium bicarbonate. Then in gel digestion was done by using 150 ng of sequencing grad trypsin in 30 μL of 50 mM ammonium bicarbonate. Extraction buffer containing 1% trifluoroacetic acid and 2% acetonitrile was used to extract cleaved peptides in each sample. The peptide mixture was added to matrix solution (2,5*-*dihydroxybenzoic acid ) for sample-matrix cocrystallization. Peptide Calibration Standard II (from Bruker, USA) was used for calibration. Resulted spectrums were analyzed for peptide mass fingerprinting (PMF) with matrix-assisted laser desorption-mass spectrometry (MALDI–TOF–TOF/MS) in a time-of-flight mass spectrometer (Ultraflex III, Bruker). Laser shots (n = 1000) at intensity between 40 and 60% were collected and summarized using the FlexControl v3 software by Bruker. Mascot Software v2.0 (Matrix Sciences, London,UK) was used to match peptides and protein automatically. All extraneous peaks, such as trypsin autodigests, matrix and keratin peaks were removed^17^. Cysteine carbamidomethylation and methionine oxidation were set as fixed and variable modifications, respectively. The peptide masses were then compared with the theoretical peptide masses of all proteins from Rattus using the SWISS-PROT and NCBI databases based on *p* < 0.05 threshold. Some spots resulted two peptides and only one of them is selected based on the score, molecular weight and pI value. Totally for a protein to be confirmed: (a) the assignment must be based on four or more *y*- or *b*-series ions, (b) the protein molecular mass must be consistent with the gel migration data and (c) protein pI value should also be considered. (Tables S1–S4).

### Western blot

Ubiquitin carboxyl-terminal hydrolase L1 (PGP 9.5), Transgelin 3, ADP-ribosyl cyclase 1 (ADPRC1), Calreticulin, lysophosphatidic acid receptor 3 (LPAR3) and synaptosomal associated protein (SNAP 25) were selected for the protein quantitation to be validated by western-blot analysis on the three basis: their role as central nodes in the molecular network, at least threefold changes and commercial availability of antibodies. Western blotting was carried out according to the method of Towbin et al.^20^. Briefly, the proteins were separated by SDS-PAGE and actively transferred onto PVDF membrane at 140 V for 1.5–2 h in the transfer buffer^17^. The membrane was washed four times in TBST (50 mM Tris, pH 7.5, 150 mM NaCl, 0.05% Tween 20) and then blocked with 5% BSA in TBST for overnight at 4 °C. The membrane was washed again and probed with the primary specific antibody (1:2000) (anti-Transgelin 3, sc-103293 and anti-ADPRC1, sc-15362 from SANTA CRUZ Biotechnology, USA and anti-synaptosomal associated protein 25 (ab108990) anti-calreticulin (ab92516) and anti-PGP 9.5 (ab8189) from Abcam, USA and anti-lysophosphatidic acid receptor 3 (322,745) from Biocompare company) for 2 h in blocking buffer at room temperature. Subsequently, the membrane was washed four times with TBST and then incubated with a secondary related IgG (1:5000) (Thermo Scientific) in TBST for 1 h at room temperature. After this step, the membrane was again washed four times with TBST. β-Actin (1:1000) (Cell Signaling Technology) was used as a housekeeping control. Bands containing rat hippocampus proteins were visualized using an ECL detection system according to the manual. The densities were calculated through ImageJ 1.46r; Java 1.6.0_20 software. The expression levels of proteins in both hippocampi of controls and 3 rat models of epilepsy were compared using one-way analysis of variance (ANOVA) followed by Duncan’s Multiple Range Test. Results were showed as mean ± S.E.M. Different letters (a-h) above the columns indicate significant difference between the control and epileptic groups (P < 0.05). If there is no significant difference between two bars they get the same letter.

### Histochemical analysis

A total of six rats, including three control rats and three rats from pilocarpine model of epilepsy group were used for the immunohistochemistry study. Rats were sacrificed under deep ketamine and xylazine anesthesia 4 h after the first observed seizure (3–5 stage) in chronic phase. 0.3% sodium sulphide in 0.1 M phosphate buffer (PB) was used to perfusion and 4% formaldehyde in phosphate buffer was used for fixation. The fixed brains were cryoprotected with 30% sucrose and cut using a freezing microtome at a thickness of 8 µm.

For immunohistochemistry staining, hippocampus coronal sections were treated with 0.5% Triton X-100 and 3% hydrogen peroxide for 10 min, and then with normal goat serum (1:10). The specimens were incubated with the primary antisera (anti-ADPRC1 from SANTA CRUZ Biotechnology, USA and anti-SNAP 25, anti-calreticulin and anti-PGP 9.5 from Abcam, USA) at room temperature overnight. After proper washing (3*5 min), slides incubated with anti-mouse and rabbit fluorescent secondary antibody for 2 h. After washing step nuclear staining has been done by DAPI. Slides were washed (3*2) and mounted properly for studding by fluorescent microscope.

### Bioinformatics analysis

To extract protein–protein interaction data, the molecular interaction database (IntAct: https://www.ebi.ac.uk/intact/) was used. The protein–protein interaction network of DE proteins was represented based on IntAct data. To obtain a protein interaction network, some bridge proteins have been added to the network as well as the 95 differently expressed proteins.

We have used QuickGO (https://www.ebi.ac.uk/QuickGO/), which is a web-based tool of the Gene Ontology and Gene Ontology annotation data, to assign molecular function and biological process to the differently expressed proteins (supplementary file S1). We have also checked each of these annotations against the literature. For those proteins with no GO annotation, we used the annotations that are provided in the literature. All of our analyses were done in R environment and networks have been drawn using igraph package^21^.

### Whole-cell patch-clamp recording

Young male Wistar rats (6–9 weeks old) were decapitated under ether anesthesia and their brain were quickly removed. Then the right hemisphere was dissected out in ice-cold artificial cerebrospinal fluid composed of (in mM): 238 sucrose, 2.5 KCl, 2 CaCl_2_, 2 MgSO_4_, 1 NaH_2_PO_4_, 26.2 NaHCO_3_ and 11 d-glucose equilibrated to a pH of 7.3–7.4 with 95% O_2_ and 5% CO_2_ (290–300 mOsm) and 400-μm thick transverse slices containing entorhinal cortex and hippocampus were prepared using a vibroslicer (Leica VT 1200 s, Leica Microsystems AG, Wetzlar, Germany). Then slices immediately transferred to a Gibbs chamber containing ACSF and incubated at 32–35 °C for 60 min. Before transferring the slices to the submerged recording chamber, they were kept at room temperature (23–25 °C) for at least 20 min. Recording chamber was mounted on the stage of an upright microscope (Axioskop 2 FS MOT; Carl Zeiss, Germany). The chamber was continuously superfused with the ACSF at a rate of 4 ml/min. Hippocampal CA1 pyramidal neurons were visually identified by an infrared CCD camera (IR-1000, MTI, USA) with a 40X- water immersion objective.

Whole-cell patch-clamp recording under the current clamp mode was made from CA1 pyramidal neurons. Recording microelectrodes (1.5 mm O.D. and 0.86 mm I.D., Sutter, USA) were pulled with a horizontal puller (P-97, Sutter Instrument, USA) and filled with intracellular solution containing (in mM): 115 K-gluconate, 20 KCl, 10 HEPES, 2 EGTA, 10 disodium-phosphocreatine, 2 MgATP and 0.3 NaGTP. pH was adjusted to 7.25–7.30 and osmolality was in the range of 285–290 mOsm. The tip resistance of microelectrode in bath was 4–6 MΩ. Pipette capacitance compensation and bridge balance were carried out. Series resistance was compensated by 80%. Signals were acquired via a Multiclamp 700B amplifier and digitized with a Digidata 1440 A/D converter (Molecular Devices, CA, USA). Signals were filtered and digitized at 10 kHz. Data were saved on a PC for offline analysis using pClamp 10 software.

### Epileptiform activity induction

Epileptiform activity was induced in hippocampal slices by perfusion of recording chamber with a low Mg^2+^/high-K^+^ ACSF for 15 min. The low Mg^2+^/high-K^+^ ACSF contained (in mM): 118 NaCl, 11 KCl, 1 NaH_2_PO_4_, 25 NaHCO_3_, 10 D-Glucose, 2 CaCl_2_, 0.5 MgSO_4_ (pH = 7.3–7.4, 290–300 mOsm). In control slices, after baseline recording for 2 min, the low Mg^2+^/high-K^+^ ACSF was applied to slices for 15 min and neuronal spiking was recorded. Then the slices were washed out by ACSF for 10 min. To assess the effects of 2-Aminoethyl diphenylborinate (50 μM) and 8-bromo-cADP (100 μM) on epileptiform activity induced by the low Mg^2+^/high-K^+^ ACSF, after a 2 min baseline recording, a group of slices were subjected to ACSF containing drugs for 10 min and the low Mg^2+^/high-K^+^ ACSF induced epileptiform activity was recorded in the presence of each drug and compared with that of induced in the absence of each drug.

## Results

### Hippocampus proteome in control and the rat models of epilepsy

**Figure 1:**
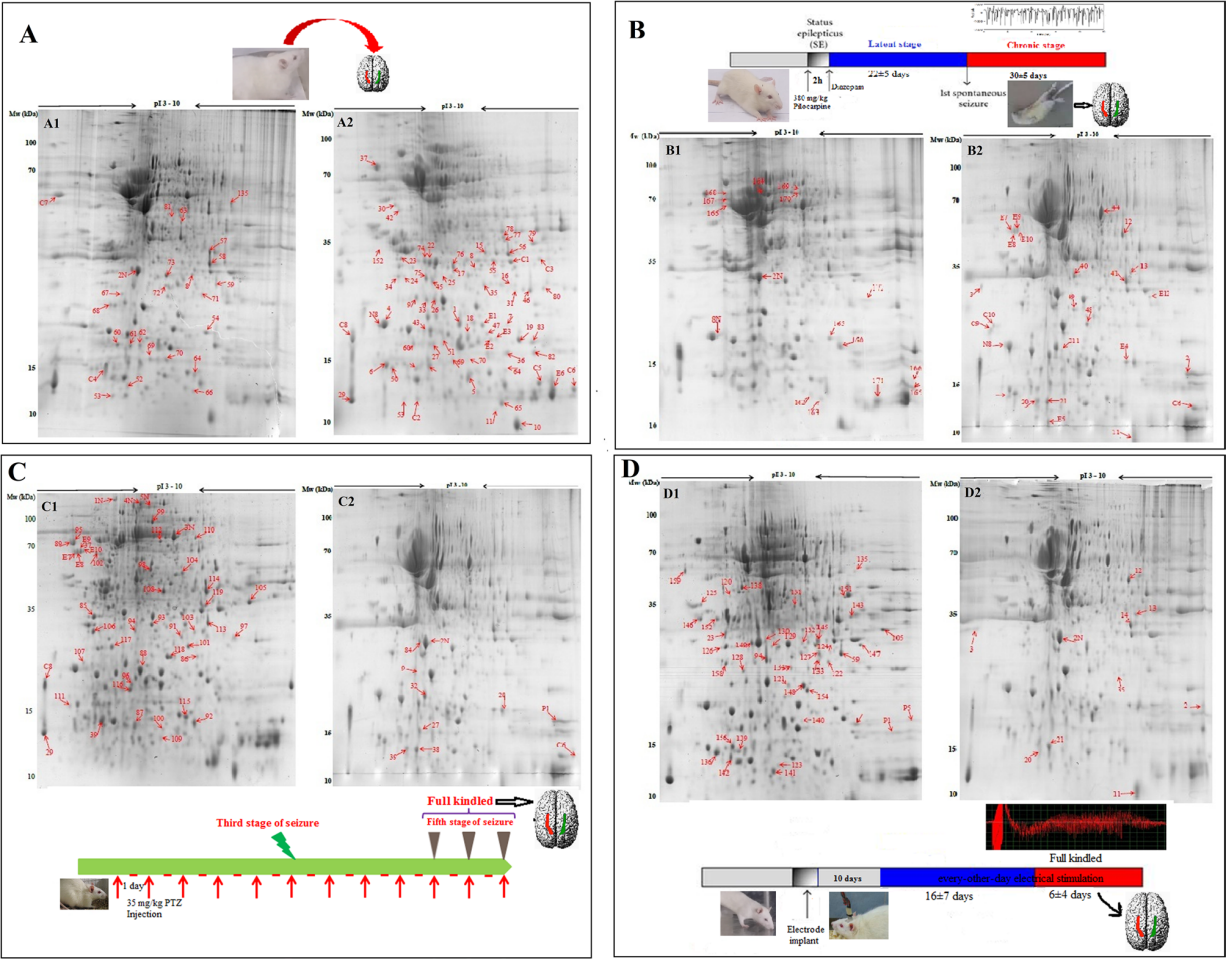
2-DE analysis of the hippocampus proteome. The timeline of both control and epileptic tissue sampling is shown in each panel. One milligram of total proteins of each sample was separated by 2-DE on a pH 3–10 linear IPG strip in the first dimension and on a 12.5% SDS–PAGE gel in the second dimension. Representative gels from control tissues were showed in (**A**): the left (**A1**) and the right hippocampus (**A2**); panel (**B**) is related to pilocarpine model: the left (**B1**) and the right hippocampus (**B2**); panel (**C**) showed gels related to PTZ kindling model: The left (**C1**) and the right hippocampus (**C2**) and panel (**D**) showed proteome profile of left (**D1**) and right (**D2**) hippocampus related to electrical kindling model. Differently expressed spots were analyzed by ImageMaster 2D Platinum v7.0 and showed by arrows.

**Figure 2:**
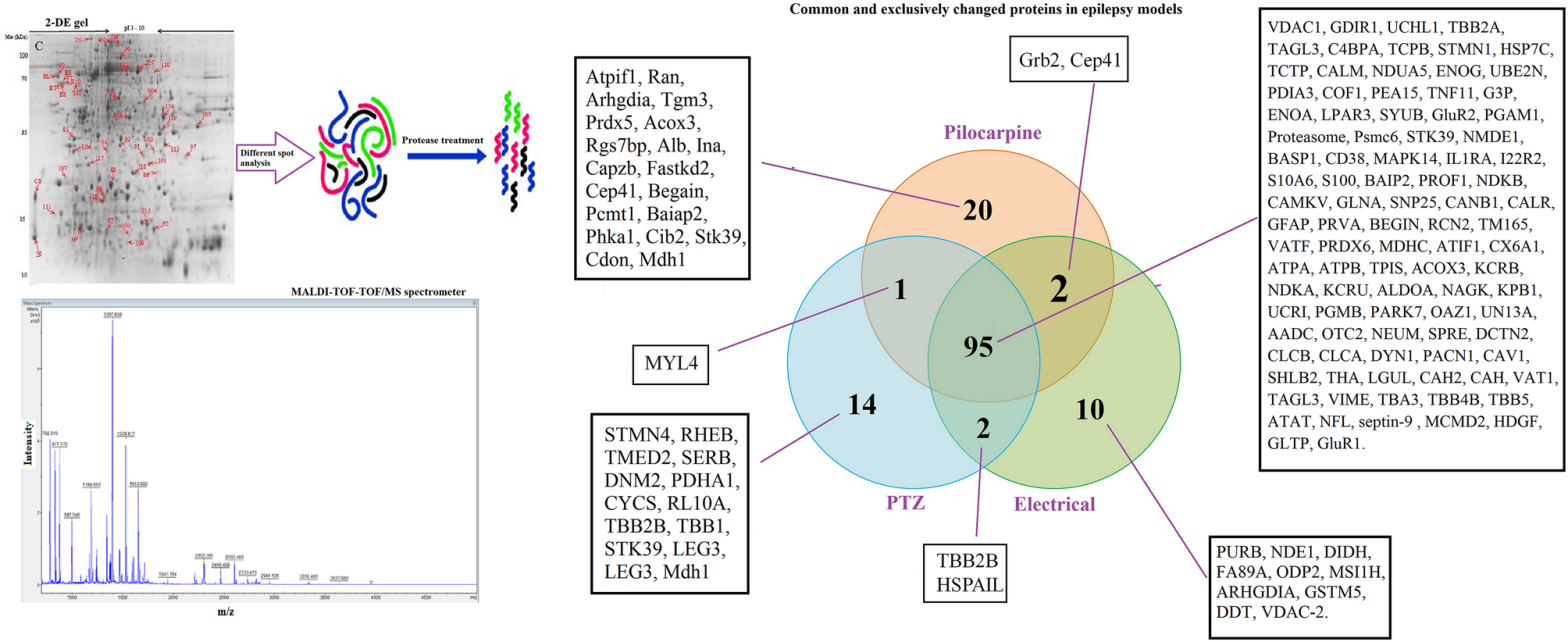
The process of detection and recognition of different spots. Differently expressed spots in each epilepsy model rather than control were visualized and recognized by MALDI–TOF–TOF/MS spectrometer method after trypsin digestion. Commonalities and differences of changed proteins categorized between three models as a Venn diagram. The short name of each identified protein for each group is indicated.

To clarify the molecular differences between wildtype rats and the rat models of epilepsy, proteomic profiling of the rat hippocampus from the kindling and pilocarpine models of epilepsy was performed. We constructed proteome profile of the right and the left hippocampi in all experimental groups using a broad range of IPG strips from pH 3–10. The ImageMaster 2D Platinum v7.0 gel analysis software confirmed 1300 ± 150 spots on each gel (Fig. 1). A gel with maximum number of spots and the acceptable resolution was chosen as a master gel and then spots of the other gels were coordinated automatically by software and corrected manually. The proteome of each rat model was compared separately with that of control group considering laterality. As shown in Fig. 2, 144 differently expressed spots were identified across groups (Tables S1–S3). Among them, 95 spots were common for both hippocampi across groups (Table S4). Selected spots were then isolated, exposed to in-gel trypsin digestion and finally recognized with MALDI–TOF–TOF/MS. The identified proteins were classified into the biological pathways was conducted using QuickGO (https://www.ebi.ac.uk/QuickGO/), a web-based tool for Gene Ontology and Gene Ontology annotation data.

#### *Signaling network analysis*

**Figure 3:**
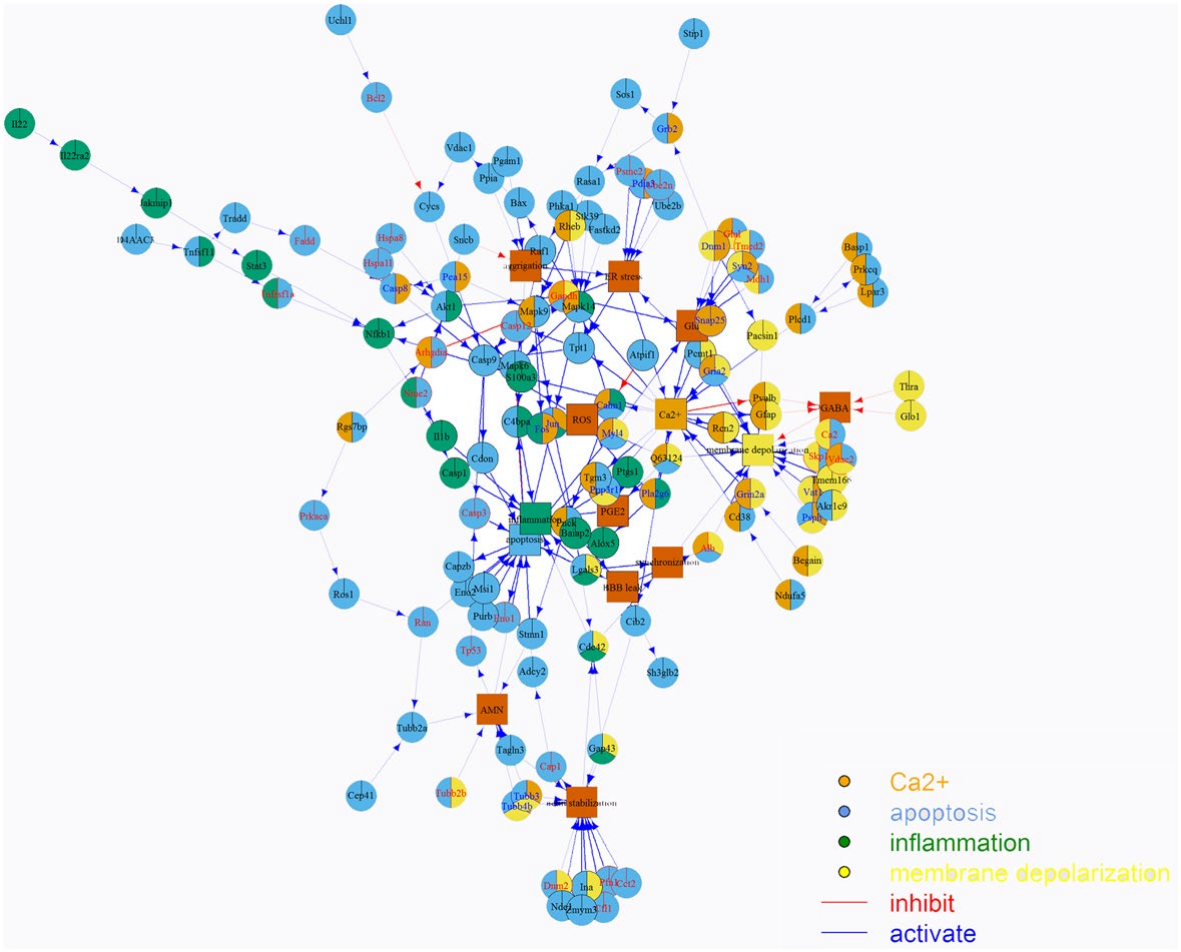
Molecular interaction network with 139 nodes consists of 125 proteins and 14 functions. The interaction numbers are 196 which are classified as two types: inhibiting (blue) and activating (red). Network consists of 95 detected proteins and some bridge nodes. Expressed proteins are clarified in 4 of 14 functions: Ca^2+^ influx, apoptosis, inflammation and membrane depolarization.

To study the interplay of proteins, protein complexes, signaling pathways and network modules for the 95 differentially expressed proteins across the three rat models of epilepsy, the signaling pathway analysis using QuickGO (https://www.ebi.ac.uk/QuickGO/), as a web-based tool of the Gene Ontology and Gene Ontology annotation data was conducted (Fig. 3). This molecular network consists of 139 nodes (125 proteins and 14 functions) and 196 inhibitory and activatory interactions.

Some of the identified proteins including calreticulin, glutamate NMDA receptor subunit epsilon-1 (NMDE1), glutamate AMPA receptor R1 and R2, ADPRC, LPAR3 and others have a role in calcium homeostasis (Table 1). The observed apoptotic and necrotic cells in the hippocampal tissue samples obtained from rat models of epilepsy (Fig. S4B) can be caused by a reduced expression level of UCH-L1 and a significant increase in expression levels of apoptosomal microtubular network organizers (transgelin-3, cofilin-1, profiling-1 and GAP43) triggering a biochemical cascade that leads to the acute neuronal death.

**Table 1:** The list of proteins differently expressed in three models of epilepsy in comparison with control that involved in calcium homeostasis pathways.

Spot number	Name of protein	Fold change	pI/Mw	Accession no	Score
34	S100 calcium binding protein A3	+ 3.1	4.86/12.6	EDM00546	61
C2	CaM kinase-like vesicle-associated protein	+ 2.8	5.37/54.1	Q63092	57
8	Protein-glutamine gama-glutamyl transferase E (TG)	+ 2.7	6.46/77.2	D4A5U3	78
3	Synaptosomal-associated protein (SNAP)	− 2.3	4.66/23.3	P60881	105
163	NADH dehydrogenase [ubiquinone] 1 alpha subcomplex subunit 5	+ 3.1	6.84/13.4	Q63362	76
25	ADP-ribosyl cyclase 1 (ADPRC1)	+ 3.2	8.83/34.4	Q64244	53
50	Calcineurin subunit b type 1 (Protein phosphatase 2B regulatory subunit 1 or CaN)	+ 2.6	4.64/19.3	P63100	74
2N	Calreticulin (calregulin, CRP55, CaBP3, ERp60)	+ 2.4	4.33/48	P18418	54
164	Glial fibrillary acidic protein (GFAP)	+ 3.4	5.35/49.9	P47819	208
139	Parvalbumin alpha (PV)	− 3.2	5.00/12	P02625	89
75	Lysophosphatidic acid receptor 3 (LPAr3)	+ 3.0	9.49/40.3	Q8K5E0	54
34	Brain-enriched Guanylate Kinase-associated Protein (BEGAIN)	+ 3.4	5.88/67	O88881	53
69	Reticulocalbin-2 (RCN2)	− 3.1	4.27/37.4	Q62703	126
123	Transmembrane protein 165	− 2.1	6.97/34.8	Q4V899	53
64	Glutamate [NMDA] receptor subunit epsilon-1	+ 3.8	6.59/165	Q00959	167
167	Glutamate receptor 1 (GluR1)	+ 2.7	7.46 / 101	P19490	68
12	Glutamate synthase (GS)	+ 3	6.64/42.3	P09606	97
62	AMPA-selective glutamate receptor 2 (GluR2)	+ 3.2	7.12/98.7	P19491.2	78
160	Calcium and integrin-binding family member 2 (RL10A_RAT)	+ 2.1	9.94/24.8	P62907	72
6	Calmodulin	+ 3.1	4.09/16.8	P62161	56
43	Phosphorylase b kinase regulatory subunit alpha	+ 3.2	5.60/139	Q64649	56
47	Synaptic vesicle membrane protein vat-1	+ 2.9	6.17/43.1	Q3MIE4	134
42	Clathrin light chain B	− 2.0	4.56/25.1	P08082	57

#### Immuno-blotting experiments confirm 2-DE results

**Figure 4:**
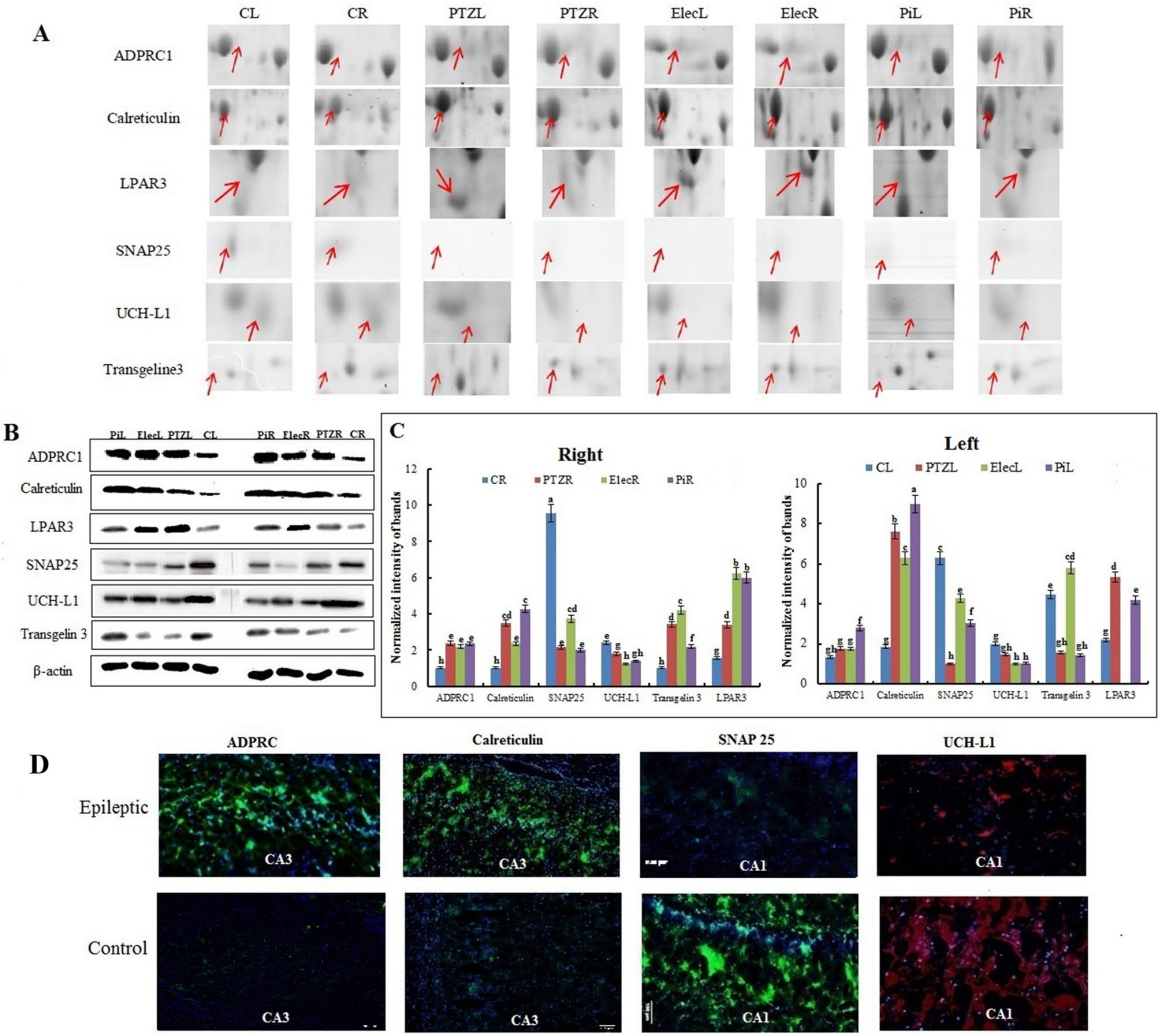
Immunoblotting analysis. (**A**) 2-DE gel images of six protein spots of interest denoted by an arrow in each panel. Each panel shows an expanded 2-D gel view and ranked from the left to the right: the left (CL) and the right hippocampus (CR) of the control group; the left (PTZL) and the right hippocampus (PTZR) of the PTZ group; the left (ElecL) and the right hippocampus (ElecR) of the electrical kindling group; and the left (PiL) and the right hippocampus (PiR) of the pilocarpine group. (**B**) Western blot analysis validates the differential expression of ADPRC1, Calreticulin, SNAP 25, UCH-L1, LPAR3 and transgelin 3 in the control and the epileptic groups. Beta-actin was used for normalization. (**C**) The intensity of bands was quantified by ImagJ software. The data were expressed as mean ± S.E.M. The letters above the columns (a-h) indicate significant differences (P < 0.05) using the Duncan’s multiple range test so, data that showed with the same letter don’t have significant differences. (**D**) Representative photographs of immune-histochemical analysis of the expression levels of the ADPRC1, the calreticulin, the SNAP 25 and the UCH-L1 in control and epileptic groups.

The expression levels of ADPRC, calreticulin, UCH-L1, SNAP 25, transgelin 3 and LPAR3 were examined in both hippocampi across groups by western blotting. Higher expression levels were observed for ADPRC, calreticulin, LPAR3 and transgelin 3 across the 3 rat models of epilepsy (Fig. 4). The expression of these proteins were higher in the right hippocampus than those in the left hippocampus of control animals, but their asymmetric expression was not significant after a seizure, except the expression of calreticulin, that was higher in the left hippocampus when compared to the right hippocampus across the 3 rat models of epilepsy (Fig. 4). A decrease in the expression levels of UCH-L1 and SNAP 25 was also seen across the 3 rat models of epilepsy with no laterality (Fig. 4).

To clarify the tissue distribution of the identified proteins in the hippocampus, ADPRC, calreticulin, SNAP 25 and UCH-L1 were selected for immune-histochemical analysis. An increase of ADPRC in both the CA1 and the CA3 regions and of calreticulin in the CA3 region was found across the 3 rat models of epilepsy. However, a significant reduction of both UCH-L1 and SNAP 25 expression levels was observed in the CA1 across the 3 rat models of epilepsy (Fig. 4). Thus, these findings indicate the locally dependent changes of the identified proteins in the hippocampus using 2-DE and immunoblotting analysis.

#### Epileptic seizures modify hippocampal asymmetry

The protein expression profiles of both hippocampi were studied to determine whether seizures would change the asymmetry of protein expression. Analyzing the proteins that were involved in Ca^2+^ homeostasis showed upregulation of the ADPRC, LPAR3, AMPA and NMDA receptor expression, while UCH-L1 was weakly expressed in the control left hippocampus. Also, transgelin 3 expression level in the left native hippocampus was more than that of the right native hippocampus (Fig. 4B). We found significant changes in the asymmetric expression of proteins after a seizure including UCH-L1 expression across the 3 rat models of epilepsy (Fig. 4B). A higher ADPRC production in the left hippocampus was seen following epileptic seizures. An increase in transgelin 3 expression level was seen on the both hippocampi across the 3 rat models of epilepsy with different fold. Therefore, findings indicated changes in the laterality of protein expression following epileptic seizures.

### Inhibition of calcium influx suppresses in vivo and in vitro seizures

**Figure 5:**
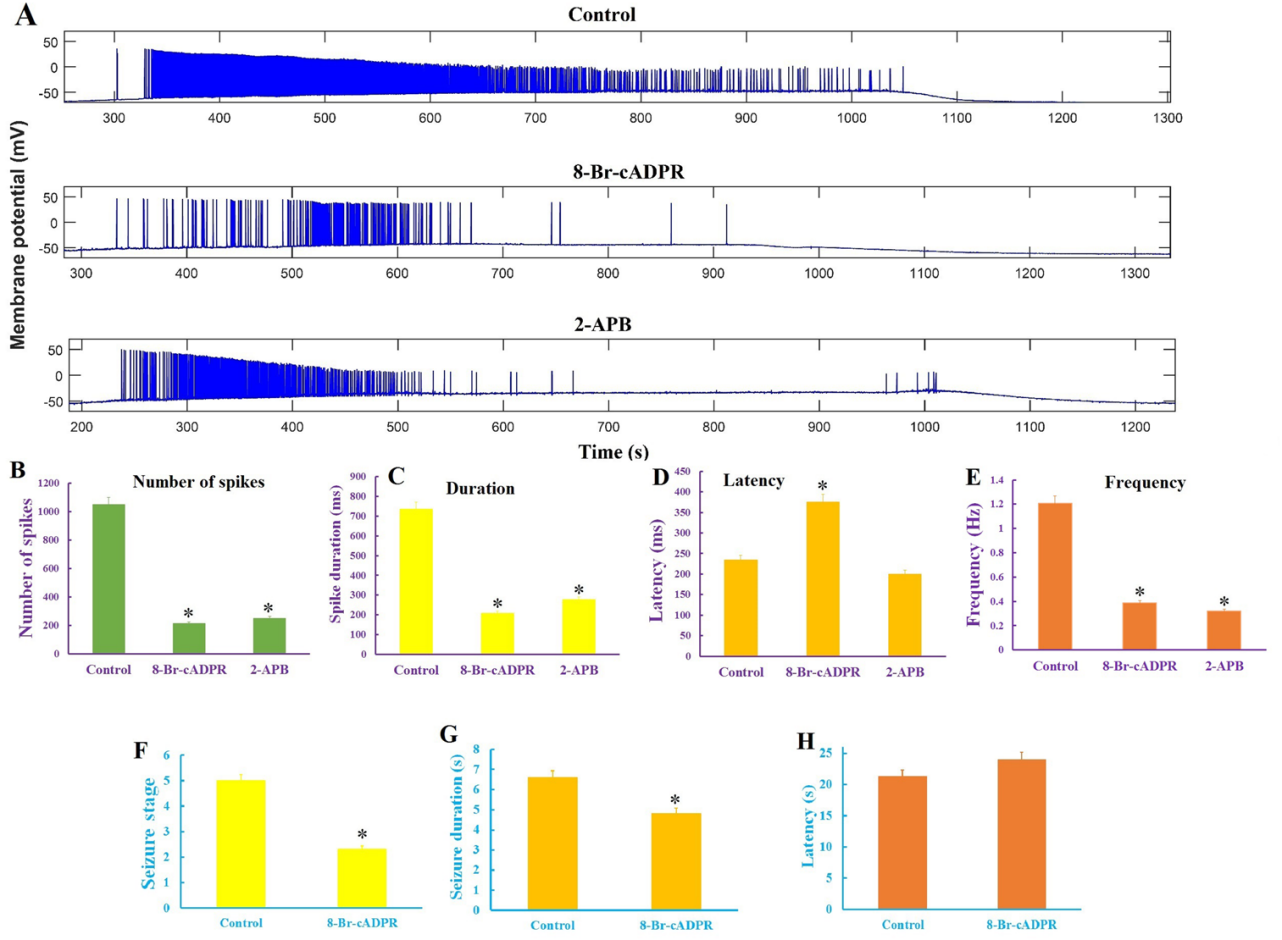
Blocking of calcium influx inhibits seizure in both in vivo and in vitro seizure models. (**A**) Suppression of the high-K^+^/low-Mg^2+^ induced epileptiform activity in the CA1 region of the hippocampal slice in the presence of 8-Br-cADPR and 2-APB. Inhibition of epileptiform discharges by reducing of the spiking numbers (**B**), spiking duration (**C**), onset latency of spiking (**D**) and spiking rate (**E**), in the presence of the both Ca^2+^ channels inhibitors, particularly in the presence of 8-Br-cADPR. 8-Br-cADPR suppresses epileptic seizures in the PTZ-kindled rats (**F**) as indicated by a significant decrease in seizure duration (**G**) and an insignificant effect on seizure latency (**H**). Data were expressed as mean ± S.E.M. Differences between groups were analyzed by one‑way analysis of variance (ANOVA). *Represents a significant difference as compared to control group (P < 0.05).

**Figure 6:**
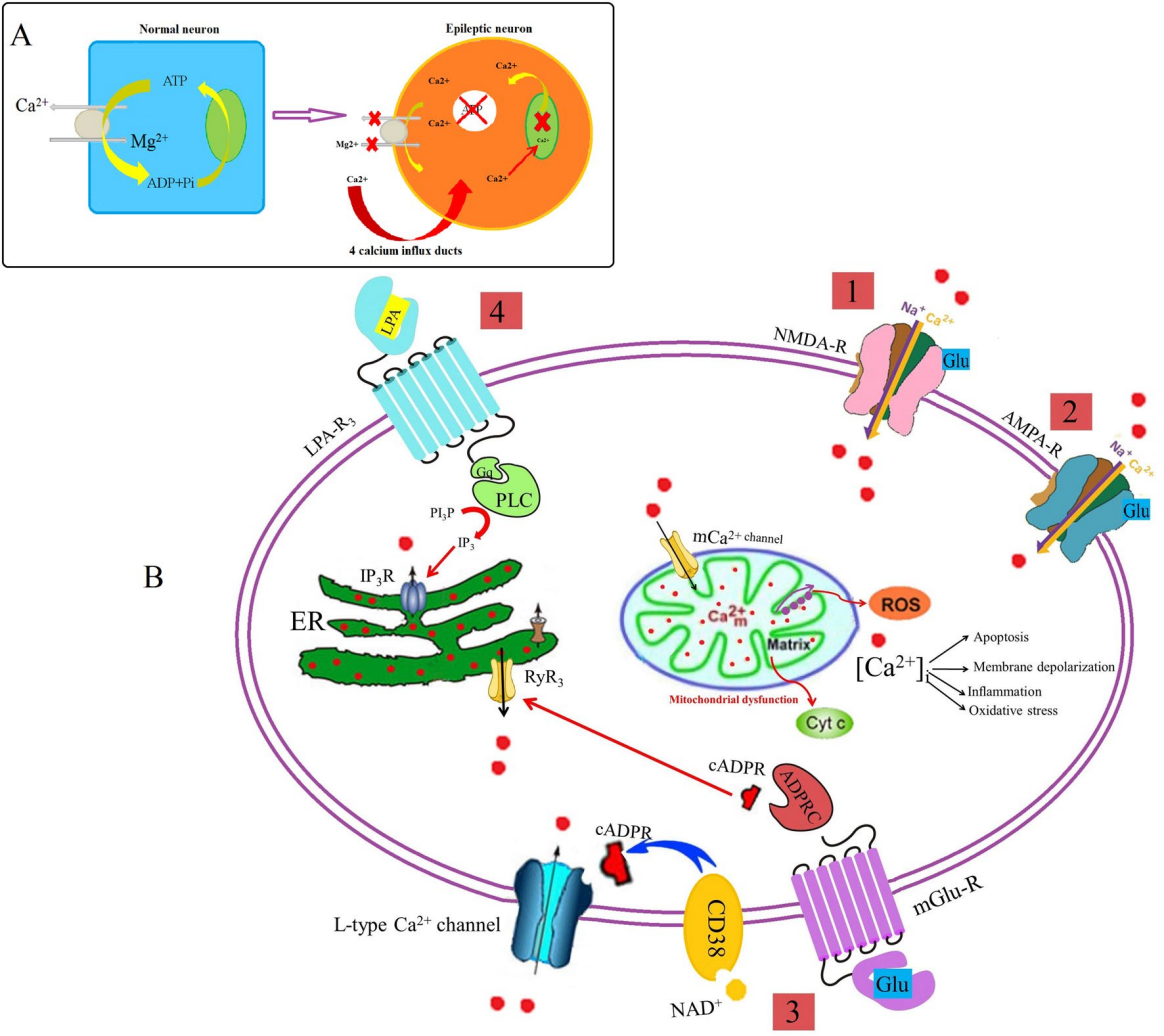
Schematic overview of intracellular Ca^2+^ cycling. (**A**) The most prominent homeostatic event in epileptic hippocampus is the accumulation of intracellular calcium. (**B**) General scheme of Ca^2+^ influx pathways indicating the disrupted pathways in epileptic hippocampus: 1. NMDA glutamate receptor 2. AMPA glutamate receptor 3. Calcium influx activated by ADPRC as a second messenger, and 4. Ca^2+^-release channels from endoplasmic reticulum including LPAR3 receptor pathway.

As shown in Fig. 3, seizures were associated with changes in the regulators of calcium influx. Acute treatment of hippocampal CA1 pyramidal neurons with 8-Br-cADPR and 2-APB, which inhibited calcium influx through ADPRC and LPAR pathways (inhibitor of IP3 receptor and TRP channel), caused a significant reduction in spik numbers (1047.5 ± 120 in control slices, 214 ± 32 and 250 ± 38 in the 8-Br-cADPR- and the 2-APB-treated slices, respectively) and neuronal firing rate (1.207 ± 0.1 Hz in control slices, 0.388 ± 0.14 Hz and 0.320 ± 0.09 Hz in the 8-Br-cADPR- and the 2-APB-treated slices, respectively; Fig. 5) in epileptiform discharges that were imposed by low-Mg^2+^/high-K^+^ ACSF in hippocampal slices. In addition, 8-Br-cADPR significantly put off (1376.73 ± 78.23 s) the onset of epileptiform discharges as compared to the control group. Interestingly, injection of 8-Br-cADPR (2 µmol/kg) inhibited tonic-colonic seizure (the reduction of seizure stage 5 to stage 2 or 3) that was accompanied by a significant reduction in seizure duration (Figs. 5F, 6H) in the PTZ-kindled rats.

## Discussion

To date, a large body of scientific evidence indicates the involvement of numerous proteins and signaling cascades in epilepsy^4, 6^. Changes in the expression of immediate-early genes, receptors which recognize excitatory and inhibitory neurotransmitters and ion channels have been recognized in epileptic patients and animal models of epilepsy^11, 22^. Available drug therapies can only target symptoms rather than causes in epileptic patients^23^. Herein proteomic profiling of the rat hippocampus from the kindling and pilocarpine models of epilepsy was performed to unravel epileptogenic insults-induced changes in protein regulatory networks. A total of 144 differently expressed proteins in both left and right hippocampi by two-dimensional electrophoresis coupled to MALDI–TOF–TOF/MS were identified across the 3 rat models of epilepsy. Based on network analysis the majority of differentially expressed proteins were associated with Ca^2+^ homeostasis. Changes in ADPRC, Calreticulin, UCH-L1, SNAP 25, transgelin 3 and LPAR3 were probed by Western blot analysis and validated using immunohistochemistry. Inhibition of calcium influx using 8-Br-cADPR and 2-APB, which act via the ADPRC and LPAR3 pathways, respectively, attenuated epileptic seizures.

Proteomic analysis of 95 common differentially expressed proteins across three rat models of epilepsy (Fig. 2), were categorized based on their biological functions. 90 differentially expressed proteins were qualified for network analysis based on protein–protein interactions reported in database and literature. As indicated in the resulted network (Fig. 3), nods of calcium signaling network have a central role in the regulation of cellular processes including inflammation, apoptosis, oxidative stress, metabolism rate, membrane depolarization and plasticity. We showed an increase in the components of Ca^2+^ influx pathway including the glutamate NMDA/AMPA receptor, ADPRC1, LPA3R and calreticulin across the 3 rat models of epilepsy (Fig. 6) As previously confirmed, pathophysiological changes in the brain could be induce by small changes in calcium channels or their regulators^9, 10^. It has been reported that the higher expression level of the NMDA receptor leads to an increase in calcium influx in the presence of elevated glutamate across due to the up-regulation of glutamate synthase and Krebs cycle enzymes as previously reported^24^. Clinical studies also confirmed the elevated synaptic glutamate as main excitatory neurotransmitter, in the epileptic brain^25^. An increase in the AMPA glutamate receptor is associated with epileptic seizures. The glutamate binds to the AMPA receptors and causes opening of ion channels, which allows Ca^2+^ and Na^+^ to influx across the cell membrane^26^. The ADPRC1 was also increased across the 3 rat models of epilepsy. This enzyme facilitates the production of cADPR from NAD^+^^27^ and the ADP ribose involves in regulation of calcium homeostasis^27, 28^. Up-regulated LPAR3 increases intracellular Ca^2+^ through the 4th pathway as indicated in Fig. 6. Lysophosphatidic acid binds to LPA3R and produces inositol three phosphate (IP3), which in turn binds to own receptors on the ER surface and causes the release of Ca^2+^ from intracellular store^29^. The overexpression of calreticulin in the hippocampal CA3 across the 3 rat models of epilepsy also regulates Ca^+2^ uptake and release by the ER^30^. Therefore, results showed an excessive increase in Ca^2+^ content of the epileptic brain from various resources can trigger some pathophysiological aspects of epileptic seizure^9, 10^.

Among mentioned components of Ca^2+^ influx pathway, the role of the NMDA and the AMPA receptors in epileptogenesis and seizure-induced brain damage has extensively been investigated and in the treatment of epilepsy the clinical trials of topiramate and perampanel, which can block these receptors have been done^31, 32^. The role played by ADPRC and LPAR3 components of Ca^2+^ influx pathways in epilepsy has not yet been demonstrated. We found that inhibition of the ADPRC and LPAR using 2-APB and 8-Br-cADPR suppressed epileptiform activity as evidenced by increasing the latency to the spike developing and decreasing the spikes number and frequency (Fig. 5). 2-APB and 8-Br-cADPR inhibited epileptic spikes after about 400 s. In-vivo studies also confirmed the anticonvulsive effect of 8-Br-cADPR so that its intravenous injection could prevent the occurrence of stage 5 seizures in PTZ kindled rats (Fig. 5) indicating seizures attenuation by inhibition of these targets. An improving effect of 8-Br-cADPR on the ropivacaine-induced convulsions in rats has recently been reported^33^. Inhibition of ADPRC can also ameliorate acute lung injury and arrhythmia^34, 35^. By considering accompaniment of epilepsy and seizure, some of the identified proteins are likely to be expressed as a result of seizure but not epilepsy. Therefore, these proteins may presumably be involved in non-epileptic seizures and not have a role in epileptic seizures. In addition, comparison of the hippocampus proteome from control and epileptic animals revealed a significant increase in some proteins involved in dendrite genesis and axon outgrowth, which can enhance neuronal plasticity as has previously been discussed^36^.

## Conclusion

Proteomic profiling of the rat hippocampus from the kindling and pilocarpine models of epilepsy could yield a sophisticated insight into pathophysiology of epileptic seizures which is critical to identify epilepsy biomarkers and its therapeutic targets. Overall, the present study demonstrated how groups of proteins work in concert with each other to keep the brain hyperexcitable following seizure induction. Based on experimental evidence, alterations in various processes such as apoptosis, inflammation, energy metabolism, dopamine content, oxidative stress, networks remodeling and membrane electrophysiology can be induced by epilepsy, so drug design strategies should be directed against multiple targets. Among differently expressed proteins, a set of proteins involved in the Ca^2+^ homeostasis appears to be most important due to their central roles in other processes. Since inhibition of Ca^2+^ flux by NMDA and AMPA receptors antagonizing could not quench seizures incidence in clinical cases, so overexpression of proteins regulating cellular Ca^2+^ homeostasis, particularly ADPRC and LPAR, could be considered as potential targets to establish effective polypharmacy in the epilepsy cure. This study introduces involvement of ADPRC and LPAR3 in epilepsy so that inhibition of these components significantly suppressed seizure and epileptiform activity in full kindled rats and hippocampal slice, respectively. Therefore, ADPRC and LPAR could be new potential targets in calcium regulatory network for drug discovery and design in treating epileptic seizures. Considering the similarity between the rat models of TLE and the human TLE, some of these identified and quantified proteins could be underlined in the epilepsy pathology and considered as potential candidates for the treatment of epilepsy in future.

## Supplementary Information

Supplementary Information 1.

Supplementary Information 2.

Supplementary Information 3.
