# Supplementary material for: Proteomic profiling of the rat hippocampus from the kindling and pilocarpine models of epilepsy: potential targets in calcium regulatory network
Source: Sci Rep. 2021 Apr 15;11:8252. doi: 10.1038/s41598-021-87555-7 (PMC8050094; doi:10.1038/s41598-021-87555-7)
Supplement: Supplementary file 1 — Supplementary Information 1. [file 41598_2021_87555_MOESM1_ESM.pdf]

SUPPORTING INFORMATION

Proteomic profiling of the rat hippocampus from the kindling and pilocarpine models of epilepsy: Potential targets in calcium regulatory network

Leila Sadeghi[1, 2], Albert Anatolyevich Rizvanov [3], Bahareh Dabirmanesh[1], Ilnur Ildusovich Salafutdinov [3], Mohammad Sayyah[4], Amir Shojaei[5], Javad Zahiri[6], Javad Mirnajafi-Zadeh[5], Babak Khorsand [7], Khosro Khajeh*[1], Yaghoub Fathollahi*[5]

[1]Department of Biochemistry, Faculty of Biological Science, Tarbiat Modares University, Tehran, Iran.

[2]Department of Animal Biology, Faculty of Natural Science, Tabriz University, Tabriz, Iran

[3]Department of Genetics, Kazan Federal University, Kazan, Russian Federation.

[4]Department of Physiology and Pharmacology, Pasteur Institute of Iran, Tehran, Iran.

[5]Department of Medical Physiology, Faculty of Medical Science, Tarbiat Modares University, Tehran, Iran.

[6]Department of Biophysics, Faculty of Biological Science, Tarbiat Modares University, Tehran, Iran.

[7]Department of Computer Engineering, Faculty of Engineering, Ferdowsi University of Mashhad, Mashhad, Iran

Short title: Hippocampus proteome, calcium homeostasis and seizures

Table S-1: The list of proteins that showed different expression exclusively in PTZ kindling model in comparison with control hippocampus.

Row no.	Spot number	Match ID	Name	Fold change	pI/Mw	Accession no	Score	Function
1	111	920	Myosin Light Chain 4	+3.4	4.96/21.2	P17209	98	Calcium ion binding protein
2	107	8100	Stathmin-4	+2.5	5.76/22	P63043	124	Stathmin interacts with two molecules of dimeric α,β-tubulin to form a tight ternary complex called the T2S complex. Mice without stathmin have deficiency in innate and learned fear. They lack motivation for retrieving pups and are unable to choose a safe location for nest-building.
3	29	7621	GTP-Binding Protein Rheb	+2.3	5.65/20.4	Q62639	85	Activates the protein kinase activity of mTORC1, and thereby plays a role in the regulation of apoptosis.
4	96	862	Transmembrane Emp24 Domain-Containing Protein 2	+2.5	5.08/22.7	Q63524	93	Involved in vesicular protein trafficking. Mainly functions in the early secretory pathway but also in post-Golgi membranes.
5	117	778	Phosphoserine phosphatase	+3.2	5.47/24.9	Q5M819	134	Catalyzes the last step in the biosynthesis of serine from carbohydrates. The reaction mechanism proceeds via the formation of a phosphoryl-enzyme intermediates
6	110	248	Dynamin-2 (DNM2)	-3.1	7.02/98.2	P39052	90	High expression of this protein may be manifested increasing of the neurotransmitters (excitatory) releasing in epileptic models. The behavioral study revealed that inhibiting dynamin 1 increased the latency time of the first seizure and decreased the frequency and severity of the seizures These results indicate that the altered level of dynamin 1 may contribute to the development of epileptic seizures.
7	91	752	Pyruvate dehydrogenase E1 component alpha Subunit (PDHA1)	+3.6	8.49/43 212.55	P26284	67	The pyruvate dehydrogenase complex catalyzes the overall conversion of pyruvate to acetyl-CoA and CO2, and thereby links the glycolytic pathway to the tricarboxylic cycle.
8	100	986	Cytochrome c (CYCS)	+3.5	9.61/11.5	P62898	57	Electron carrier protein. Plays a role in apoptosis.
9	160	PTZ11	Calcium and integrin-binding family member 2 (RL10A_RAT)	+2.1	9.94/24.8	P62907	72	This protein is similar to that of KIP/CIB, calcineurin B, and calmodulin. It is calcium-binding regulatory protein that interacts with DNA-dependent protein kinase catalytic subunits (DNA-PKcs).
10	93	716	Tubulin beta-2B chain (TBB2B_RAT)	+2.3	4.78/50	Q3KRE8	185	Overexpression of the Tubulin beta causes ER stress lead to apoptosis. Mutation and changes of concentration cause seizure and mental retardation due to changing in cell proliferation program.
11	161	PTZ12	Tubulin beta-2C chain	+2.0	4.79/49.8	AAH60597	182	Overexpression of the Tubulin beta causes ER stress lead to apoptosis. Mutation and changes of concentration cause seizure and mental retardation due to changing in cell proliferation program.
12	162	PTZ13	beta-3 tubulin (gi|348529924)	+2.1	4.82 / 504	NP_640347	180	Overexpression of the Tubulin beta causes ER stress lead to apoptosis. Mutation and changes of concentration cause seizure and mental retardation due to changing in cell proliferation program.
13	86	801	Galectin-3 (LEG3_RAT)	+3.1	8.59 / 27.2	P08699	157	Galactose-specific lectin which binds IgE. May mediate with the alpha-3, beta-1 integrin the stimulation by CSPG4 of endothelial cells migration.
14	2PTZ	1543	Malate dehydrogenase	+2	6.16/36.5	O88989	60	This cytoplasmic enzyme converts malate to pyruvate in glycolysis pathway. (MDH) is an enzyme that reversibly catalyzes the oxidation of malate to oxaloacetate using the reduction of NAD^+ to NADH. Previous studies showed decreasing of the MDH activity in rats that injected by PTZ may be due to reduced expression.
15	311	311	Myosin Light Chain 4	+3.6	4.96/21.2	P17209	72	Calcium ion binding protein

Table S-2: The list of proteins that showed different expression exclusively in Electrical kindling model in comparison with control hippocampus.

Row no.	Spot number	Match ID	Name	Fold change	pI/Mw	Accession no	Score	Function
1	178	1724	Transcriptional Activator Protein Pur Beta	-3.1	5.33/33.4	Q68A21	183	DNA binding transcription factor that have role in cell differentiation.
2	178	1723	Nuclear Distribution Protein Nude Homolog 1	-2.1	5.32/38.5	Q9ES39	98	Required for centrosome duplication and formation and function of the mitotic spindle.
3	177	1623	3-Alpha-Hydroxy steroid Dehydrogenase (3α-HSD)	-3.1	6.67/37	P23457	92	the enzyme can accomplish diverse functions: as quinone reductase, as an aromatic alcohol dehydrogenase
4	176	1743	Protein FAM89A	+2.2	5.35/18.5	Q6Q0N2	51	Unknown
5	73	1732	Dihydrolipoamide Acetyltransferase (DLAT)	+3.2	5.70/58.7	P08461	62	The pyruvate dehydrogenase complex catalyzes the overall conversion of pyruvate to acetyl-CoA and CO2, and thereby links the glycolytic pathway to the tricarboxylic cycle.
6	173	1735	RNA-binding protein Musashi homolog 1 (MSI1)	+2.1	7.71/39.1	Q8K3P4	152	RNA binding protein that regulates the expression of target mRNAs at the translation level. Regulates expression of the NOTCH1 antagonist NUMB. Binds RNA containing the sequence 5'-GUUAGUUAGUUAGUU-3' and other sequences containing the pattern 5'-[GA]U1-3AGU-3'.
7	175	1724	Rho GDP-dissociation inhibitor 1(ARHGDIA)	-2.6	5.12/23.4	Q5M9P6	86	It plays an essential role in control of a variety of cellular functions through interactions with Rho family GTPases, including Rac1, Cdc42, and RhoA. RhoGDI is frequently overexpressed in human tumors and chemo-resistant cancer cell lines, raising the possibility that RhoGDI might play a role in the development of drug resistance in cancer cells. It inhibits apoptosis pathway.
8	165	1634	Glutathione S-transferase Mu 1 (GSTM5)	+3.1	8.42/25.7	P04905	139	Conjugation of reduced glutathione to a wide number of exogenous and endogenous hydrophobic electrophiles. The olfactory GST may be crucial for the acuity of the olfactory process.
9	171	1624	D-dopachrome Decarboxylase (DDT)	+2	6.15/13.0	P80254	75	Tautomerization of D-dopachrome with decarboxylation to give 5,6-dihydroxyindole (DHI).
10	174	1625	synapsin-2 (SYN2)	+4.1	8.73/63.4	Q63537	69	Neuronal phosphoprotein that coats synaptic vesicles, binds to the cytoskeleton, and is believed to function in the regulation of neurotransmitter release. May play a role in noradrenaline secretion by sympathetic neurons (By similarity).
11	173	1662	Voltage-dependent anion-selective channel protein 2 (VDAC-2)	+3.2	7.44/31.7	P81155	54	Forms a channel through the mitochondrial outer membrane that allows diffusion of small hydrophilic molecules. The channel adopts an open conformation at low or zero membrane potential and a closed conformation at potentials above 30-40 mV.
12	44	1654	Heat shock 70 kDa protein 1L (HSPAIL)	+2.9	5.91/70.5	P55063	95	A cochaperone that organizes other chaperones and heat shock proteins (HSPs). It induced in stressor condition.
13	74		Centrosomal protein of 41 kDa (TSGA14 or Cep41)	-2.1	5.44/37	Q4KM37	58	This protein is essential to glutamylation of the tubulins. Glutamylation defects related to Joubert Syndrome. Mutation of the gene responsible to this protein causes Joubert Syndrome and autism; both of them are nervous system mutagenic disorder. Impaired social interaction and communication, repetitive and stereotyped behavior, congenital ataxia, hypotonia and developmental delay are common events in down regulation or mutation condition.

Table S-3: The list of proteins that showed different expression exclusively in pilocarpine model in comparison with control hippocampus.

Row no.	Spot number	Match ID	Name	Fold change	pI/Mw	Accession no	Score	Function
1	4	2529	ATPase inhibitor	+2.5	9.6/12.2	Q03344	51	Energy metabolism: inhibition of ATP hydrolyses for maintenance of ATP homeostasis in the conditions that neurons have to use more ATP.
2	16	C14	GTP binding nuclear protein, Alpha tubulin	New spot	7.01/24.4	P62828	72	GBP increased in response to the oxidative stress and lead cells to the apoptosis
3	45	7415	Rho GDP dissociation inhibitor protects cancer cells against apoptosis	-3.2	5.10/23.4	Q5XI73	71	It plays an essential role in control of a variety of cellular functions through interactions with Rho family GTPases, including Rac1, Cdc42, and RhoA. RhoGDI is frequently overexpressed in human tumors and chemo-resistant cancer cell lines, raising the possibility that RhoGDI might play a role in the development of drug resistance in cancer cells. It inhibits apoptosis pathway.
3	8	2441	Protein-glutamine gama-glutamyl transferase E (TG)	+2.7	6.46/77.2	D4A5U3	78	Tissue transglutaminase posttranslationally modifies proteins by transamidation of specific polypeptide bound glutamines (formation of the N6-(L-isoglutamyl)-L-lysine isopeptide, resulting in cross-linking polypeptide chains). TG contributes to the pathology of several neurodegenerative conditions including Alzheimer’s disease and Huntington’s disease
4	36	2522	Peroxiredoxin-5, mitochondrial	+4.1	7.94/22.2	Q9R063	68	Peroxiredoxin-5 is a member of the peroxiredoxin family of antioxidant enzymes, which reduce hydrogen peroxide and alkyl hydroperoxides. Overexpression of this protein showed activation of defense system in response to harsh oxidative stress.
5	154	2539	Peroxisomal acyl-coenzyme A oxidase 3 (ACOX 3)	+2.3	8.44/78.4	Q63448	148	Energy metabolism: Clinical abnormalities include hypotonia, psychomotor retardation, hepatomegaly, facial dysmorphism and seizure resembling those described in peroxisome deficient Zellweger syndrome.
6	25	2481	Regulator of G-protein signaling 7-binding protein (RGS7BP)	+2.7	8.84/28.9	Q5FVH8	45	RGS proteins are multi-functional, GTPase-accelerating proteins that promote GTP hydrolysis by the alpha subunit of heterotrimeric G proteins, thereby inactivating the G protein and rapidly switching off G protein-coupled receptor signaling pathways.
7	115	4PTZ	Serum albumin	+4.1	6.09/68.7	P02770	51	Increasing of the serum albumin caused by dysfunction of the blood–brain barrier (BBB) that is a common finding during seizures or following epileptogenic brain injuries, and experimentally induced BBB opening promotes seizures both in naive and epileptic animals.
8	1N	1N	Alpha I internexin	+3.2	5.20 / 56.1	P23565	64	It belongs to NF-L. Alpha I internexin has important role in cell proliferation and differentiation such as other neurofilament.
9	40	7282	F-actin-capping protein subunit beta (CAPZB)	+2.1	5.69/30.6	Q5XI32	109	CAPZB binds to the end of actin filament and cause calcium independent growth of filamentous. Therefore it has a important role in cell shape and growth. It seems CAPZB increasing cause activation of glials that is biomarker of brain injuries such as glial acidic protein and cofiline.
10	64	2708	Fast kinase domain-containing protein 2 (FASTKD2)	+2.5	8.88/77.7	Q5M7V7	51	Fas-activated serine/threonine phosphoprotein (FAST) localized in the mitochondrial inner compartment that inhibits apoptosis by inhibition of TIA-1 promotes increased expression of cIAP and XIAP to inhibit apoptosis. this protein produced in harsh condition and interact with respiratory complex and TIA-1 for modulate the expression of inflammatory mediators. FASTKD family members are essential components of mitochondrial respiration that may modulate the energy balance in cells exposed to adverse conditions of cytochrome c oxidase activity
11	74		Centrosomal protein of 41 kDa (TSGA14 or Cep41)	-2.1	5.44/37	Q4KM37	58	This protein is essential to glutamylation of the tubulins. Glutamylation defects related to Joubert Syndrome. Mutation of the gene responsible to this protein causes Joubert Syndrome and autism; both of them are nervous system mutagenic disorder. Impaired social interaction and communication, repetitive and stereotyped behavior, congenital ataxia, hypotonia and developmental delay are common events in down regulation or mutation condition.

12	34	7476	Brain-enriched Guanylate Kinase-associated Protein (BEGAIN)	+3.4	5.88/67	O88881	53	BEGAIN is PSD component associated with the core complex of PSD-95/SAP90 and SAPAP. Resulted complex have different domains such as guanylate kinase and SH3 so it can interact with different kind of proteins in different signaling pathway. This complex can interact with NMDA and potassium channel and recruits them in membrane.
13	8	8	Protein-L-isoaspartate (D-aspartate) o-methyl transferase	-2.3	7.13/24.6	P22062	61	Its chaperon that recognizes and catalyzes the repair of damaged L-isoaspartyl and D-aspartatyl groups in proteins. PIMT tends to act on proteins that have been non-enzymatically damaged due to age. By performing this repair mechanism, the enzyme helps to maintain overall protein integrity. Therefore down expression of this cause ER stress lead to the apoptosis.
14	69	2650	Brain specific angiogenesis inhibitor I associated protein (Baiap2)	+3	9.10/59.2	Q6GMN2	154	This protein quenchs inhibitor and involved in interaction of angiogenesis (as an cytoplasmic protein) and cell membrane that is important to angiogenesis. Inflammation chronic epilepsy resulting from new blood vessels formation and a weakened blood-brain-barrier (BBB) function. Increasing of angiogenesis showed activation in epileptic brain that approved increasing of other related proteins
15	43	7499	Phosphorylase b kinase regulatory subunit alpha	+3.2	5.60/139	Q64649	56	Its involve in energy metabolism. The enzyme phosphorylase kinase (PhK) activates liver glycogen phosphorylase and brain glycogen phosphorylase in response to neuronal and hormonal stimuli and thus is a key regulatory enzyme in glycogen breakdown. Calcium and other excitatory agents such as acetylcholine (Pi agonist) cause more activity and more expression of this protein (high energy demand of neuronal cells).
16	43	43	Calcium and integrin binding family member 2	+2.8	4.45/21.6	Q568Z7	148	This protein is similar to that of KIP/CIB, calcineurin B, and calmodulin. It is calcium-binding regulatory protein that interacts with DNA-dependent protein kinase catalytic subunits (DNA-PKcs).
17	43	43	STE 20/SPS-1 related proline-alanin rich protein kinase	+3.2	5.92/60	O88506	52	This gene encodes a serine/threonine kinase that is thought to function in the cellular stress response pathway. The kinase is activated in response to hypotonic stress, leading to phosphorylation of several cation-chloride-coupled cotransporters.
18	E7	E7	Cell-adhesion molecule-related/down-regulated by Oncogenes (CDON)	+2.1	6.53/136.2	O35158	51	CDON behaves as a SHH dependence receptor: it actively triggers apoptosis in the absence of SHH. The pro-apoptotic activity of unbound CDON requires a proteolytic cleavage in its intracellular domain, allowing the recruitment and activation of caspase-9
19	2PTZ	1543	Malate dehydrogenase	+2	6.16/36.5	O88989	60	Energy metabolism: Critical enzyme in ATP biosynthesis pathway and Glutamate synthesis as an important excitatory neurotransmitter
20	311	311	Myosin Light Chain 4	+3.6	4.96/21.2	P17209	72	Calcium binding protein

Table S-4: The list of the 95 differently expressed proteins in epileptic hippocampus.

Row no.	Spot number	Match ID	Name	Fold change	pI/Mw	Accession no	Score	Function
1	13	C13	Glyceraldeyde-3-phosphate dehydrogenase	+3	8.14/35.8	P04797	83	Energy metabolism: glycolysis enzyme
2	E9	2051/2061	Growth associated protein 43	+2.5	4.61/23.6	AAI27503	176	Glutamate can increase expression of Gap43 in excitation conditions and this protein changes releasing of the neurotransmitters
3	E7	2051/2061	Cysteine deoxygenase type 1 (COD)	-2.5	5.98/23	P21816	52	COD catalysis the oxidation of l-cysteine to cysteine sulfinic acid. This protein has cell survival role by localization of the DJ-1 protein in mitochondria.
4	5	7581	Interlukin-22 receptor subunit alpha 2	+3.5	9.19/26.7	Q7TNI4	56	It binds to inhibitors and antagonists of inflammation pathway so can quench inflammation.
5	109	1PTZ	C4-binding protein alpha chain	New spot	7.06/62.2	Q63514	73	One of the C1 complex members that has main effect on phagocytosis and cause uncontrollable release of the pre-inflammatory agents. Inflammatory and apoptotic effects.
6	E10	2051/2061	T-complex protein 1	+2.3	5.5/59.5	Q5XIM9	60	A molecular chaperon that consume ATP and fold actin and tubulin. Increasing of this protein demonstrated in schizophrenia.
7	88	2581	Thyroid hormone receptor protein	New spot	6.91/55.1	P63059	151	Inter nuclear protein binds to Triiodothyronine and cause excitatory status in body such as high blood pressure that take place in seizures.
8	54	C13	Transgelin-3	+3.7	6.84/22.5	P37805	133	It directly binds to P53 and actin and regulates expression of the MMP9 gene. Transgelin is required in agonist-induced smooth muscle contraction and is involved in cell migration. It has tumour suppressive functions in certain cells.
9	12	2200	Glutamate synthase (GS)	+3	6.64/42.3	P09606	97	This enzyme regulates protein, GABA, osmotic regulations synthesis pathway and its dysfunction cause seizure
10	9	MO	Lactoglutathione lyase (glycoxalase I)	+2.4	5.12/20.8	Q6P7Q4	53	It is a neuromodulator or chaperone that catalyzes isomerization of the hemithioacetal. High expression of this enzyme increases MG concentration and inhibits of GABA pathway lead to seizure.
11	E8	2051/2061	Neuromodulin	+2.5	4.61/23.6	P07936	59	Neuromodulins are neuropeptides or hormones can change releasing of the neurotransmitters so alter speed of transmission through nerves and between them.
12	106	2484	Tubulin beta-2A chain	New	4.78/49.9	P85108	148	Overexpression of the Tubulin beta causes ER stress lead to apoptosis. Mutation and changes of concentration cause seizure and mental retardation due to changing in cell proliferation program.
13	24	23	Glycolipid transfer protein (GLTP)	+3.1	6.9/23.7	B0BNM9	42	This protein transmits the glycolipids and not phospholipids between cell membrane and organism membranes so it control cell size in cell cycle and has role in cell proliferation
14	C5	451	Protein unc-13 homolog A	Lost	5.1/196	Q62768	52	It involved in maturation of vesicles so it has important role in neurotransmitter releasing in nervous system
15	19	24	Nucleoside diphosphate kinase B	+3.2	6.9/17.3	P19804	123	It modulates neuronal cell proliferation, differentiation, and neurite outgrowth. However, a role of NDPK in neurodegenerative diseases has not been reported yet. Its level decreased in patients with Alzheimer's disease (AD) and Down syndrome (DS).
16	78	16	Peroxiredoxin-6	+3	5.64/24.8	O35244	150	This is a ubiquitous family of antioxidant enzymes that also control cytokine-induced peroxide levels and thereby mediate signal transduction in mammalian cells. This protein has been reported to be up-regulated in the kainic acid-induced rat model of CSE, as well as in animal models of amyotrophic lateral sclerosis
17	105	615	V-type proton ATPase subunit E1	+3.5	8.44/26.1	Q6PCU2	85	It is a subunit of V-ATPase so involve in energy metabolism involve in Transferrin endocytosis and recycling, Insulin receptor recycling, Iron uptake

								and transport and Insulin receptor signalling cascade. It reduced in alzheimer's disease.
18	141	1036	V-type proton ATPase subunit F	+3	6.10/13.4	XP645434	140	It is a subunit of V-ATPase so involve in energy metabolism involve in Transferrin endocytosis and recycling, Insulin receptor recycling, Iron uptake and transport and Insulin receptor signalling cascade. It reduced in alzheimer's disease.
19	C1	C1	Triose phosphate isomerase	+4	6.89/26.8	P48500	90	This protein decreased in Electrical and PTZ models (sample C1) showed importance of energy production in Pi model, because Pi treated rats could not eat any food so lost more than 30% of their body weight so ATP production has more importance for them. Therefore enhanced expression of the energy production enzymes is critical in Pi treated rats.
20	C2	C1	CaM kinase-like vesicle-associated protein	+2.8	5.37/54.1	Q63092	57	It has an important role in transferring and releasing of the neurotransmitters from neurons calcium dependently. Therefore releasing of neurotransmitters faced with problem in down regulated cells.
21	23	26C	Ornithine decarboxylase antizyme 1 (ODC-AZ)	-3.2	6.71/25.2	P54370	61	Ornithine decarboxylase inhibitor can bind and quench a crucial enzyme in polyamines biosynthesis pathway, Polyamines regulate calcium channel so control calcium dependent releasing of neurotransmitters. Expression of ODC-AZ decreased in alzheimer disease.
22	79	S21	Voltage dependent onion-selective channel protein 1	+2.4	8.62/30.8	Q9Z2L0	162	TM (transmembrane) protein located in cell membrane and mitochondrial membrane and has crucial role in controlling of the cell size. It has important role in apoptosis due to transferring of the low molecular weight apoptotic agents (such as ATP, ADP, cytochrome c and etc). Its level changed in Alzheimer's and Down syndrome.
23	69	2650	Myotrophin	+2.5	5.27/12.9	P62775	67	This protein has a potential function in cerebellar morphogenesis, and it may be involved in the differentiation of cerebellar neurons, particularly of granule cells (it promote differentiation and inhibit cell proliferation). This protein is one of the fundamental proteins in brain development.
24	148	856	Protein DJ-1 (Parkinson-associated protein)	-4.3	6.32/20	O88767	120	It can bind to the 20s proteasome and inhibit it so can prevent degradation of semi-unfolded proteins. Expression of DJ-1 increase in stressor condition. Mutation of this gene leads to sensitivity to MPTP (oxidant agent) and Parkinson disease.
25	62	1342	AMPA-selective glutamate receptor 2<br>(GluR2)	+ 3.2	7.12/98.7	P19491.2	78	Receptor for glutamate that functions as ligand-gated ion channel in the central nervous system and plays an important role in excitatory synaptic transmission. L-glutamate acts as an excitatory neurotransmitter at many synapses in the central nervous system.
26	C	C1	Stress induced phosphoprotein 1	+3.6	6.40/62.6	O35814	192	A cochaperone that organizes other chaperones and heat shock proteins (HSPs). STIP1 Activates the ALK2-SMAD Signaling Pathways and Promotes Cell Proliferation of Cancer Cells and rescue cells from apoptosis by activation of ERK pathway.
27	45	7415	Ubiquitin carboxyl-terminal hydrolase L1	-2.9	5.14/24.8	Q00981	54	In the absence of UCH-L1, synaptic transmission at the neuromuscular junctions (NMJs) is markedly impaired. Both spontaneous and evoked synaptic activities are reduced. Loss of normal UCH-L1 activity lead to neurodegeneration in the central and peripheral nervous system
28	3	P11	Synaptosomal-associated protein (SNAP)	-2.3	4.66/23.3	P60881	105	This protein plays a pivotal role in regulating synaptic vesicle exocytosis, verbal and performance intelligence quotient, learning, and memory and spatial memory. Inhibition of hippocampal SNAP-25 results in impairment of long-term contextual fear and spatial memories and a decrease in LTP
29	21	15	Cytochrome C oxidase subunit 5a, mitochondrial	+2.1	6.07/16.1	P11240	89	Mutation in related gene cause strock (seizure disease). Previous studies showed inhibition of respiratory chain enzymes cause seizure.
30	20	15	ATP synthase subunit alpha, mitochondrial	+4	9.22/59.7	P15999	75	Energy metabolism: one of the reasons for reduction of ATP is decreasing of the functional ATP synthase may be due to oxidative stress
31	P1	P1	Sepiapterin reductase (SPR)	-2.5	5.55/28.1	P18297	53	Sepiapterin reductase catalyzes the final step in the

								biosynthetic pathway of tetrahydrobiopterin (BH4), an essential cofactor for aromatic amino acid hydrolases including tyrosine hydroxylase, the rate-limiting enzyme in dopamine synthesis. This protein increased in Parkinson Disease.
32	1	7454	Stathmin	Lost	5.75/17.3	P13668	128	Stathmin interacts with two molecules of dimeric α,β-tubulin to form a tight ternary complex called the T2S complex. Mice without stathmin have deficiency in innate and learned fear. They lack motivation for retrieving pups and are unable to choose a safe location for nest-building.
33	1	7454	Dynactin subunit 2	-3.2	5.14/44.14	Q6AYH5	46	This complex plays a critical role in cell division and the transport of materials within cells. The dynactin complex, dynein, and microtubules work together like a conveyer belt to move materials within cells. At least five mutations in related gene have been found cause Perry syndrome.
34	30	2127	Clatherin light chain A	-2.5	4.41/26.9	P08081	94	Clathrin is a major coat protein involved in sorting and retention of proteins at the late Golgi and in endocytosis from the cell surface. Decreased expression of this protein interrupted releasing of the neurotransmitter and rescuing of their receptors so interrupted all downstream process (Alzheimer).
35	105	2400	Heat shock cognate 71 kDa (HSP 70)	+3.8	5.37/70.9	P63018	129	Induction of heat shock/stress proteins is a key feature of a universal mechanism of cellular defense to injury known as the “stress response” specially in oxidative stress condition (we approved oxidative stress in epilepsy before).
36	56	2386	Carbonic anhydrase (CA)	Lost	6.89/29.11	P27139	117	CA II plays an important role in the production of CSF and regulation of its pH and ionic constituents. This enzyme required for pH balancing and low expression of that can cause long lasting seizures.
37	163	163	NADH dehydrogenase [ubiquinone] 1 alpha subcomplex subunit 5	+3.1	6.84/13.4	Q63362	76	Energy metabolism: its deficiency is associated with a wide variety of clinical signs and symptoms such as weak, loss of eye assign, seizure, brain retardation and behavioral disorders.
38	74	2413	Ubiquitin carboxyl-terminal hydrolase isozyme L1 (UCH-L1)	-2.6	5.14/24.8	Q00981	54	In the absence of UCH-L1, synaptic transmission at the neuromuscular junctions (NMJs) is markedly impaired. Both spontaneous and evoked synaptic activities are reduced. Loss of normal UCH-L1 activity lead to neurodegeneration in the central and peripheral nervous system
39	159	C7	Brain acid soluble protein 1 (BASP1)	+2.0	4.48/21.8	Q05175	51	It is one of the fundamental proteins that has role in Brain developing. The most striking property of *BASP1* is its potential to block the initiation of Myc-induced cell transformation.
40	C8	C8	Calmadulin	+2.1	4.09/16.8	P62161	56	Calmodulin (CaM) is a ubiquitous, calcium-binding protein that can bind to and regulate a multitude of different protein targets, thereby affecting many different cellular functions. CaM mediates processes such as inflammation, metabolism, apoptosis, muscle contraction, intracellular movement, short-term and long-term memory, nerve growth and the immune response. Note that calcium concentration increase in seizure. Calmodulin modulates numerous Ca^{2+}-dependent enzymes and participates in relevant cellular functions such as hyperexcitability and apoptosis.
41	25	2481	ADP-ribosyl cyclase 1 (ADPRC1)	+3.2	8.83/34.4	Q64244	53	ADPRC 1 catalyzes production of Cyclic ADP-ribose from NAD^+ that is one of the main inducers for accumulation of calcium in cytoplasm.
42	50	2633	Calcineurin subunit b type 1 (Protein phosphatase 2B regulatory subunit 1 or CaN)	+2.6	4.64/19.3	P63100	74	Calcineurin (CaN) is a ubiquitous Ca^{2+}-calmodulin (CaM)-dependent protein phosphatase that has been shown to regulate the activity of ion channels, glutamate release and synaptic plasticity. CsA and FK-506 (CaN inhibitors) have been shown to inhibit the progression of kindling in a reversible manner, suggesting that CaN may play an essential role in epileptogenesis in the kindling model.
43	131	2238	Gamma-enolase, also known as enolase 2 (ENO2) or neuron specific enolase (NSE)	+1.8	5.03/47.1	P07323	73	Energy metabolism: Enolase involved in the glycolysis. Its indicator of neurodegeneration and has been used to assess the primary injury as well as to predict outcome after various brain-damaging conditions. It increases after single seizure and SE.
44	70	2692	Ubiquitin-conjugating enzyme	-2.1	6.13/17.1	Q9EQX9	62	This enzyme decides to living and dead of protein

			E2					and its concentration increase in harsh and stress condition. this enzyme increased in neurodegenerative disease such as Alzheimer so its controlling is a potentially treatment for this ailment.
45	104	2238	Dynamin-1	+1.5	6.44/97.3	P21575	54	High expression of this protein may be manifested increasing of the neurotransmitters (excitatory) releasing in epileptic models. The behavioral study revealed that inhibiting dynamin 1 increased the latency time of the first seizure and decreased the frequency and severity of the seizures These results indicate that the altered level of dynamin 1 may contribute to the development of epileptic seizures.
46	3N	3N	Creatine kinase B-type (B-CK)specific CK in brain tissue	+2.3	5.39/42.7	P07335	63	Involved in rapid energy production that need in seizure. Previous studies showed deficiency for creatine kinase is increasing calcium removal rate from the hippocampal cells so decreased seizure probability in PTZ treated rats.
47	2N	2N	Calreticulin (calregulin, CRP55, CaBP3, ERp60)	+2.4	4.33/48	P18418	54	Calreticulin is involved with maintaining adequate calcium levels in organisms (major Ca^{2+}-binding (storage) protein in the lumen of the endoplasmic reticulum). Increasing of this protein is in agreement with increasing of the other calcium binding protein and calcium concentration in epileptic rats
48	5N	5N	Protein Disulfide isomerase	lost	5.88/56.6	P11598	53	PDIs contribute to the pathogenesis of many neurodegenerative disorders including Alzheimer's disease, Parkinson's disease and Huntington's disease. Accumulation of unfolded proteins in ER causes a condition that called ER-stress lead to the apoptosis and finally neurodegeneration.
49	42	42	Clathrin light chain B	-2.0	4.56/25.1	P08082	57	Decreased expression of this protein interrupted releasing of the neurotransmitter and rescuing of the their receptors so interrupted all downstream process
50	10	7637	Hemoglobin subunit alpha-1	+3.1	7.81/15.3	P01946	52	Brain hemoglobin has role in storage of oxygen to provide a homeostatic mechanism in anoxic conditions, which is especially important for A9 DA neurons that have an elevated metabolism with a high requirement for energy production. Increasing of this protein in hippocampus tissue showed oxygen demand in epileptic brain is more than control.
51	19	24	Cofilin-1	+2.6	8.22/18.5	P45592	90	It depolymerizes filamentous F-actin and inhibits the polymerization G-actin. Therefore increasing of the Coflin-1 cause depolymerization of the filamentous actin and affected dendritic structure and function and lead dysfunction of neurotransmitters trafficking in the cell.
52	17	17	Growth factor receptor-bound protein 2 (Grb2 or Gab 2)	-3.5	5.89/25.2	P62994	110	Contains the SH3 and SH2 domains so can bind to phosphotyrosine in Ras signal transduction pathway. This protein increases cell proliferation by mediated of MEK and SOS pathways.
53	E10	E10	Neuromodulin	+2.1	4.61/23.6	P07936	152	Neuromodulins are neuropeptides or hormones can change releasing of the neurotransmitters so alter speed of transmission through nerves and between them.
54	33	7449	Lactoylglutathione lyase (glycoxalase I)	+2.2	5.12/20.8	Q6P7Q4	101	It is a neuromodulator or chaperone that catalyzes isomerization of the hemithioacetal. It detoxifies methyl glyoxalate (2-oxo aldehyde). High expression of this enzyme increased MG concentration and inhibition of GABA pathway lead to seizure
55	6	7562	Calcineurin subunit b type 1 (Protein phosphatase 2B regulatory subunit 1 or CaN)	+3.1	4.64/19.3	P63100	63	Calcineurin (CaN) is a ubiquitous Ca^{2+}-calmodulin (CaM)-dependent protein phosphatase that has been shown to regulate the activity of ion channels, glutamate release and synaptic plasticity. CsA and FK-506 (CaN inhibitors) have been shown to inhibit the progression of kindling in a reversible manner, suggesting that CaN may play an essential role in epileptogenesis in the kindling model.
56	18	18	Nucleoside diphosphate kinase A	+2.7	5.94/17.2	Q05982	56	It modulates neuronal cell proliferation, differentiation, and neurite outgrowth. However, a

								role of NDPK in neurodegenerative diseases has not been reported yet. Its level decreased in patients with Alzheimer's disease (AD) and Down syndrome (DS)
57	102	355	Vimentin	+2.6	5.05/53.7	P31000	171	Vimentin is a kind of intermediate filament that expresses in immature astrocytes. Prolonged seizure activity markedly increases neurogenesis in the dentate gyrus of adult rats and that newly born dentate granule cells contribute to hippocampal network reorganization in the pilocarpine model of temporal lobe epilepsy. PCNA, an independent marker of mitotic activity,
58	156	P16	Astrocytic phosphoprotein PEA-15	+2.5	4.93/15	Q5U318	59	Expression of PEA-15 oscillates throughout the cell cycle, and the loss of PEA15 accelerates cell cycle progression. It induced by injuries. Previous studies showed seizures increased its expression in CA1 and CA3 about 1.95 and 2.17 fold respectively than control.
59	64	2708	Peptidyl-prolyl cis-trans isomerase A	-2.2	8.34/17.9	P10111	52	In Alzheimer’s disease, the peptidyl prolyl cis/trans isomerase Pin1 binds to phospho-Thr231 on Tau proteins and, hence, is found within degenerating neurons, where it is associated to the large amounts of abnormally phosphorylated Tau proteins. Pin1 can regulate tau oligomerization so it is one of the possibly same events that take place in epilepsy and Alzheimer’s disease or other neurodegenerative disease.
60	52	2717	Septin-9 isoform 3	+1.8	7.67/38.6	NP_001106969	58	The septins are an evolutionarily conserved family of Phosphoserine proteins involved in diverse processes including vesicle trafficking, apoptosis, remodelling of the cytoskeleton, infection, neurodegeneration, and neoplasia. Upregulation of this protein cause neuronal migration.
61	C10	C10	Tumor necrosis factor ligand superfamily member 11	+2.5	8.22/35.4	Q9ESE2	38	This protein was shown to be a dentritic cell survival factor. A number of studies in animal models indicate that seizures induce TNF expression in the brain. Up-regulating of TNF in hippocampus of epileptic rats than controls approved neurodegeneration condition (neuroiflamation) in 3 models.
62	94	732	MCM domain-containing protein 2	+1.8	8.07/76	Q5XI14	48	MCM domain-containing proteins are Helicases have major roles in genome maintenance by unwinding structured nucleic acids. They are important for genomic stability and cellular homeostasis, it seems over expression of them has a compensational role due to increasing of the DNA damages during epileptogenesis (approved by our previous studies).
63	119	16	Phosphoglycerate mutase 1 (PGAM1)	+3.2	6.67/28.8	P25113	64	It catalyzes the interconversion of 3-phosphoglycerate and 2-phosphoglycerate during glycolysis. It has been reported that this enzyme is commonly upregulated in many human cancers and other conditions that energy demand increases. PGAM1 plays an important role in coordinating glycolysis.
64	112	272	60 kDa heat shock protein, mitochondrial	+3.7	5.91/61	P63039	123	The mitochondrial chaperonin Hsp60 is a ubiquitous molecule with multiple roles, constitutively expressed and inducible by oxidative stress (approved previously). Neuronal excitability can be affected by mitochondrial alterations such as depletion of ATP, generation of ROS, elevated oxidative stress, disruption of Ca2+ homeostasis, dysregulation of excitotoxicity, and alterations in biosynthesis and metabolism of neurotransmitters, all of these conditions stimulate HSP60 overexpression.
65	75	75	Lysophosphatidic acid receptor 3 (LPAr3)	+3.0	9.49/40.3	Q8K5E0	54	Previously reported LPA binds to receptor in the hippocampus and increases intracellular Ca^{2+} ion

								levels. They have suggested that LPA may elevate the extracellular glutamate concentration, thereby stimulating AMPA/kainate receptors, and in turn, alleviating the Mg2+ block of the NMDA receptor, which finally leads to glutamate stimulation of calcium influx via NMDA receptors. They further showed that LPA induces necrosis and apoptosis in cultured primary hippocampal neurons. Previous study showed supraspinal pretreatment with LPA1 and LPA3 antagonists (VPC12249 or VPC32183) protects KA-induced neuronal cell death in the hippocampal CA3 region, suggesting that hippocampal LPA1 and LPA3 appear to be involved in KA-induced hippocampal CA3 neuronal cell death.
66	35	7399	Malate dehydrogenase (MDH), cytoplasmic	-2.3	6.16/36.5	O88989	53	This cytoplasmic enzyme converts malate to pyruvate in glycolysis pathway. (MDH) is an enzyme that reversibly catalyzes the oxidation of malate to oxaloacetate using the reduction of NAD^+ to NADH. Previous studies showed decreasing of the MDH activity in rats that injected by PTZ may be due to reduced expression.
67	97	751	Glyceraldehyde-3-phosphate dehydrogenase (GAPDH)	+3.2	8.14/35.8	P04797	51	Glyceraldehyde-3-phosphate dehydrogenase (GAPDH), long considered a traditional glycolytic protein. When this enzyme migrates to nucleus act as a transcription factor and lead to apoptosis. Expression of this protein increased in neurodegenerative disease and its localization changed also. GADH can produce protein aggregates specially after oxidative changes therefore more expression may lead to amyloid fibrils that cause neurodegeneration
68	119	16	Phosphoglycerate mutase I (PGM)	+2.5	6.67/28.8	P25113	64	Its very susceptible to oxidative damages. Nonfunctional oxidized form of phosphoglycerate mutase I can produce protein aggregates (Alzyhmer disease) due to more sensitivity of this protein therefore more expression may lead to aggregate structures that cause neurodegeneration. 2DE results showed phosphoglycerate mutase expression increased in mouse cobalt focus seizure.
69	164	152	Glial fibrillary acidic protein (GFAP)	+3.4	5.35/49.9	P47819	208	Increased expression of glial fibrillary acidic protein (GFAP) represents astroglial activation and gliosis during neurodegeneration. GFAP considered a marker protein for astrogliosis in other words its biomarker of neurotoxicity.
70	168	168	Protein kinase C and casein kinase substrate in neurons protein 1 (PACSI 1)	-3.3	5.15/50.4	Q9Z0W5	76	It plays a central role in synaptic vesicle recycling (clathrin-mediated endocytosis and participate in membrane dynamics) and interacts with huntingtin via its C-terminal SH3 domain. PACSIN1 also acts as a Tau-binding partner in regulating microtubule dynamics and is required for axonal plasticity.
71	152	653	Mitochondrial ornithine transporter 2	-4.2	9.10/30	A0A0G2K309	52	There are some similarities between HHH (deficiency of this protein) and epilepsy may be due to same involving transporter in mitochondria. It seems main transporters and proteins in mitochondria and also structure of mitochondrial membrane disturbed in epileptogenesis specially in status epilepticus of Pilocarpine model.
72	139	997	Parvalbumin alpha (PV)	-3.2	5.00/12	P02625	89	PV has efficient Ca^{2+} buffering effect and its high concentration in PV-expressing nonpyramidal cells is a prerequisite for the proficient inhibition of cortical networks (due to decreasing free or active Ca^{2+} in the cells). Previous studies showed PV deficient mice are very sensitive to PTZ injection. Decreased PV expression was found in PV+ GABAergic interneurons in schizophrenia.
73	N8	N8	β–synuclein	-2.8	4.48/14.5	Q63754	97	β–synuclei inhibits α-synuclein aggregation by facilitation of interactions of α-synuclein with fatty acids in the membrane. β-synuclein may be play a

								important role in neuroprotection by regulation of Akt signaling pathway (survival pathway). It has antioxidant effect also.
74	60	2615	Hem-binding protein	+3.4	5.05/14.7	NP_001102121	63	This protein is one member of peroxiredoxin family so has antioxidant properties. Free hem and free iron cause oxidative damages. Increasing of hem-binding protein in our study showed activation of a compensation pathway against oxidative damages that created by canvulsant methods (pilocarpine, PTZ and electrical shock).
75	C6		Caveolin-1 (Cav1)	-3.8	5.30/20.5	P41350	53	Previous studies showed in mice, one of the reasons for age-related synapse degradation is the age-related decline in the expression of the cholesterol-binding protein caveolin 1 (Cav1). This process accelerates in neurodegenerative diseases, such as Alzheimer's and Parkinson's. Cav 1 has a important role in neuroplasticity and neurotransmitter release.
76	1N	1N	Alpha internexin	-3.1	5.20/56.1	P23565	64	Alpha internexin accumulates around the amyloid fibers in Alzheimer's Disease so it's possible A-internexin plays a role in the neuronal alterations associated with B-amyloid plaque formation in Alzheimer's disease. Previous studies showed it increased in Pilocarpine model.
77	80	2345	superoxide dismutase (SOD)	+4.3	8.96/24.7	P07895	54	Previous results showed oxidative stress that lead to increased activity of SOD in epileptic patients, chemical models and also knockout animal. In the KA induction model there is an increase in ROS production and mitochondrial dysfunction.
78	70	2650	Endophilin-B2	+2.3	5.44 / 44.8	Q5PPJ9	88	Endophilin is expressed at presynaptic terminals and is involved in clathrin-coated vesicle-mediated endocytosis by binding to the endocytosis- related proteins synaptojanin and dynamin via its SH3 domain (uptake of released neurotransmitter). It increases BBB leakage.
79	34	7476	S100 calcium binding protein A3	+3.1	4.86/12.6	EDM00546	61	It can bind to calcium ion. Several molecules produced by the activation of the NFκB pathway can directly or indirectly result in intracellular Ca^{2+} release.
80	37	6851	Neurofilament light polypeptide (NF-L)	+2.8	4.62/61.3	P19527	153	Neurofilament light chain (NF-L), a component of the axonal cytoskeleton, has been shown to be elevated in the cerebrospinal fluid of patients with many types of axonal injury. Northern blotting analysis showed its expression increased in progressive myoclonus epilepsy. It's a biomarker for axonal injuries like glial acidic protein that increases in neuronal damages.
81	135	415	Proteasome<br>26S protease regulatory subunit 10B	-3.1	7.64/45.8	NP_001093979	90	Less expression of proteasome complex cause diminished protease activity in cells lead accumulation of aggregates in ER and ER stress that cause apoptosis.
82	34	7476	N-acetyl-D-glucosamine kinase (NAGK)	+4.1	5.56/37.2	P81799	87	It is a key salvage enzyme that is involved in mammalian amino-sugar metabolism. This protein is multifunctional agent: 1. enzymatic activity (phosphorylation of GlcNAc) and 2. structural function (producing new branches in dendrites)
83	158	816	Cyclin-H	+2.9	6.68/37.6	Q9R1A0	52	Its survival condition protein that increased in stress condition. The cyclin H/cdk7/Mat1 complex is found either in a free state or as a part of the transcription factor complex TFIIH.
84	69	2650	Reticulocalbin-2 (RCN2)	-3.1	4.27/37.4	Q62703	126	Reticulocalbin 2 is a calcium-binding protein located in the lumen of the ER. The protein contains six conserved regions with similarity to a high affinity Ca^{+2}-binding motif, the EF-hand. It inhibited calcineurin signaling pathway (our results showed overexpression of calcineurin) by reduction free calcium ion.
85	123	1027	Transmembrane protein 165	-2.1	6.97/34.8	Q4V899	53	May function as a calcium/proton transporter

								involved in calcium and in lysosomal pH homeostasis. Deficiency of this gene causes a complex congenital disease that accompanied with unexplained fever episodes and transient epilepsy.
86	124	1124	mitogen-activated protein kinase 14 (P38 MAP Kinase)	+2.1	5.60/41.4	NP_112282.2	85	Induces apoptosis by mediation of caspase 3 and 9.
87	64	2708	Alpha-tubulin N-acetyl transferase	-1.8	9.82/47.3	Q6MG11	54	Acetylation of the e-amino group of K40 in a-tubulin is one of different modifications that are highly conserved in ciliated organisms. These changes can destabilize cytoskeletal.
88	126	778	Profilin-1	+3.2	8.46/15	P62963	78	Profilin is an actin-binding protein involved in the dynamic turnover and restructuring of the actin cytoskeleton.<br>This restructuring of the actin cytoskeleton is essential for processes such as organ development, wound healing, and the hunting down of infectious intruders by cells of the immune system
89	126	778	Hepatoma derived growth factor (HDGF)	+2.4	4.78/26.5	Q8VHK7	54	HDGF can be found in a broad range of tissues, with highest levels in brain, lung and spleen. The expression of HDGF and HRP-3 was found to be regulated during brain development. It cause expansion of axon.
90	64	2708	Glutamate [NMDA] receptor subunit epsilon-1	+3.8	6.59/165	Q00959	167	Glutamate is the principal excitatory neurotransmitter in the brain and, as such, it inevitably plays a role in the initiation and spread of seizure activity. It also plays a critical role in epileptogenesis. It is a specific channel for calcium ion.
91	31	3210	Cytochrome b-c1 complex subunit Rieske (mitochondrial)	+3.1	9.04/29.4	P20788	94	Mitochondrial cytochrome bc1 complex (complex III) a multifunctional membrane protein complex that catalyzes electron transfer from ubiquinol to cytochrome c but also is involved in proton translocation, peptide processing and superoxide generation
92	47	7474	Synaptic vesicle membrane protein vat-1	+2.9	6.17/43.1	Q3MIE4	134	Synaptic vesicles are responsible for regulating the storage and release of neurotransmitters in the nerve terminal. *by* considering the effects of increasing the calcium current and increased VAT-1 expression its possible releasing of the acetylcholine in hippocampus improved in epileptic rat models.
93	E7	E7	Ornithine decarboxylase	+1.8	5.21/51	P09057	61	Ornithine decarboxylase inhibitor can bind and quench a crucial enzyme in polyamines biosynthesis pathway, Polyamines regulate calcium channel so control calcium dependent releasing of neurotransmitters. Expression of ODC-AZ decreased in alzheimer disease.
94	4	2529	Aromatic-L-amino-acid decarboxylase	-4.2	6.51/54	P14173	84	Dopamine synthesis
95	167	167	Glutamate receptor 1 (GluR1)	+2.7	7.46 / 101	P19490	68	Glutamate receptor and calcium channel

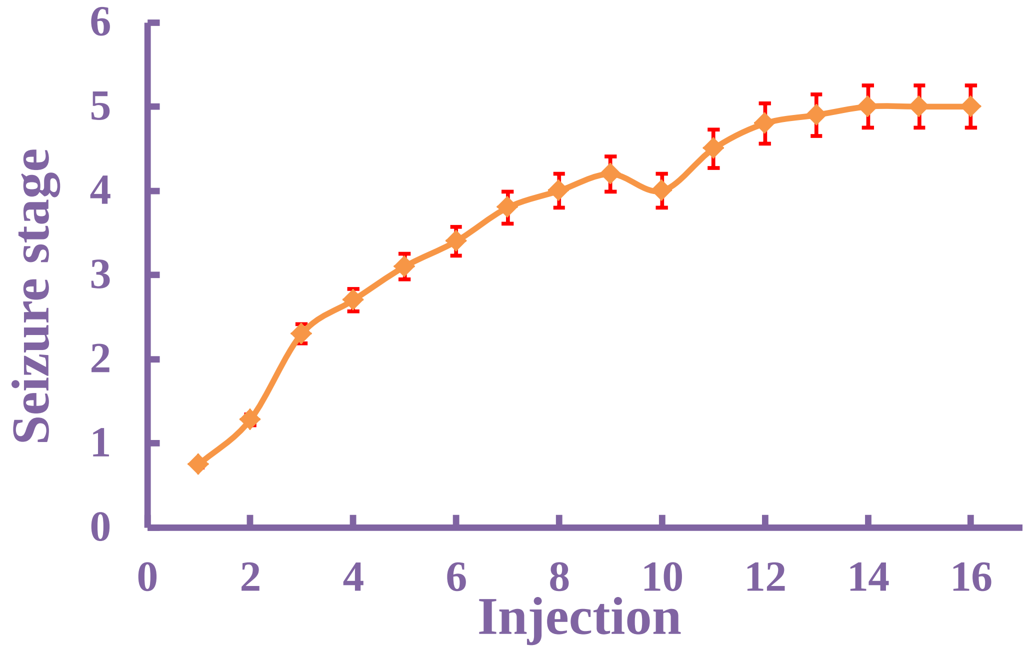

Figure S-1: seizure progressive curve in PTZ kindling model. A high proportion of rats (over 95 %) reached full kindling criterion (3 consecutive stages 4 or 5) by a total of 16 PTZ injections (35 mg/kg).

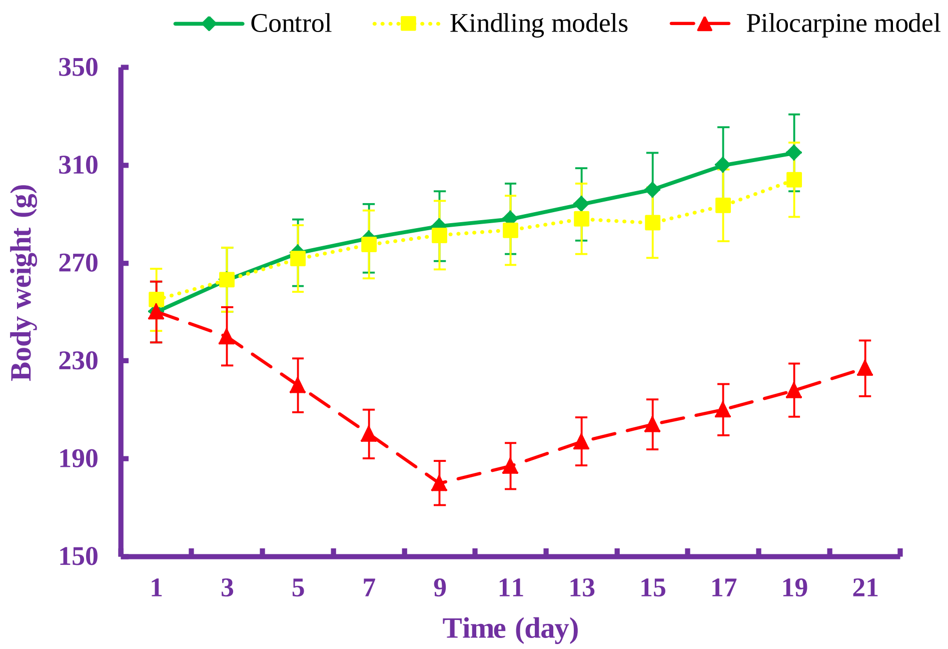

Figure S-2: Fluctuation of body weight in kindling and non-kindling (pilocarpine) model during experiment.

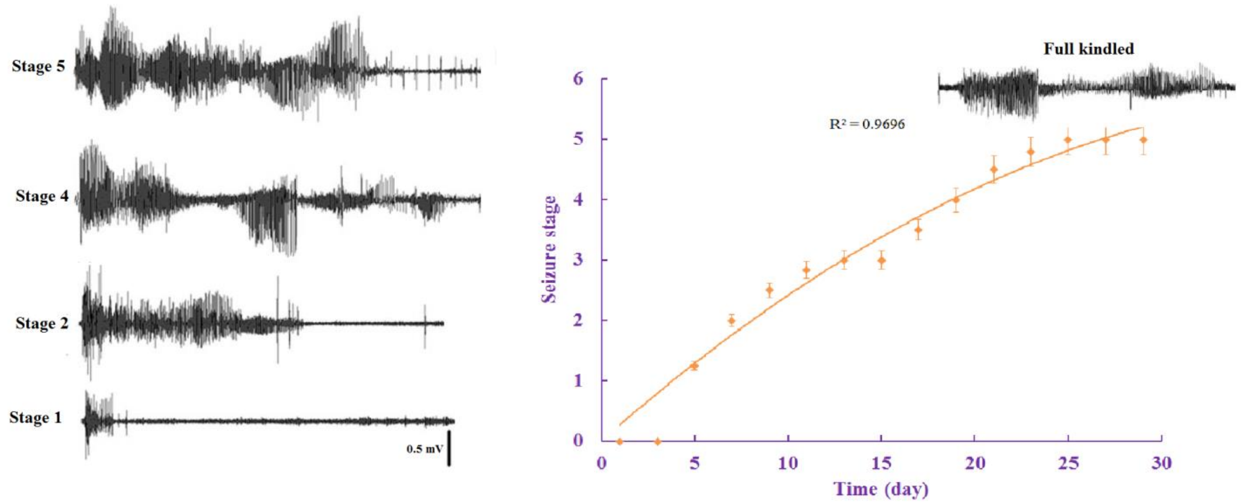

Figure S-3: Electrical kindling progress curve in rat model. The left panel shows after discharges recorded from amygdala of rats during kindling.

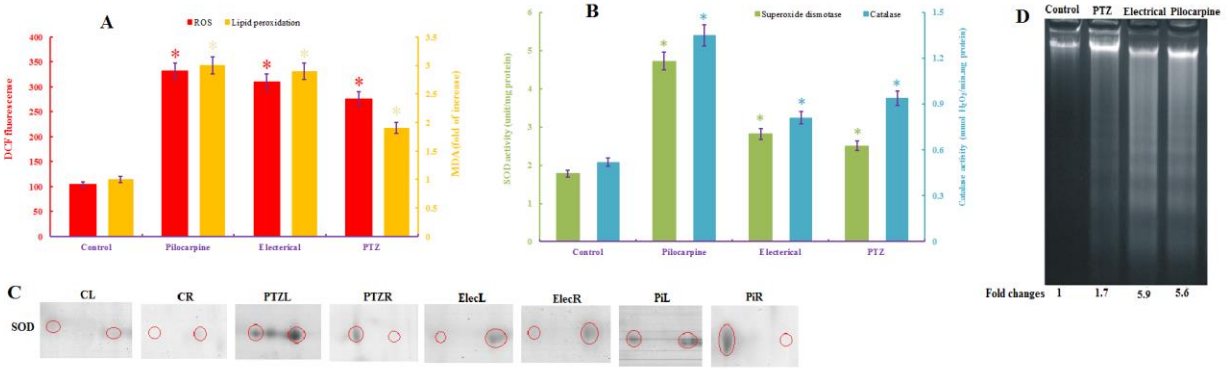

Figure S-4. Oxidative stress evaluation in epileptic rats. A) DCF fluorescence results showed ROS levels in control and epileptic rats and MDA content showed increased lipid peroxidation in epileptic rats. B) SOD and catalase activity measurements in control and three model of epilepsy showed increased activity of them that accompanied by up-expression of SOD based on proteomics data (C). Asterisk symbols showed significant changes by $P<0.05$. D) Gel electrophoresis of genomic DNA extracted from hippocampus tissues of control and epileptic rats. Fold changes of intensity between 100 to 150 bp fragments for each sample compared with control were showed below the gel image.

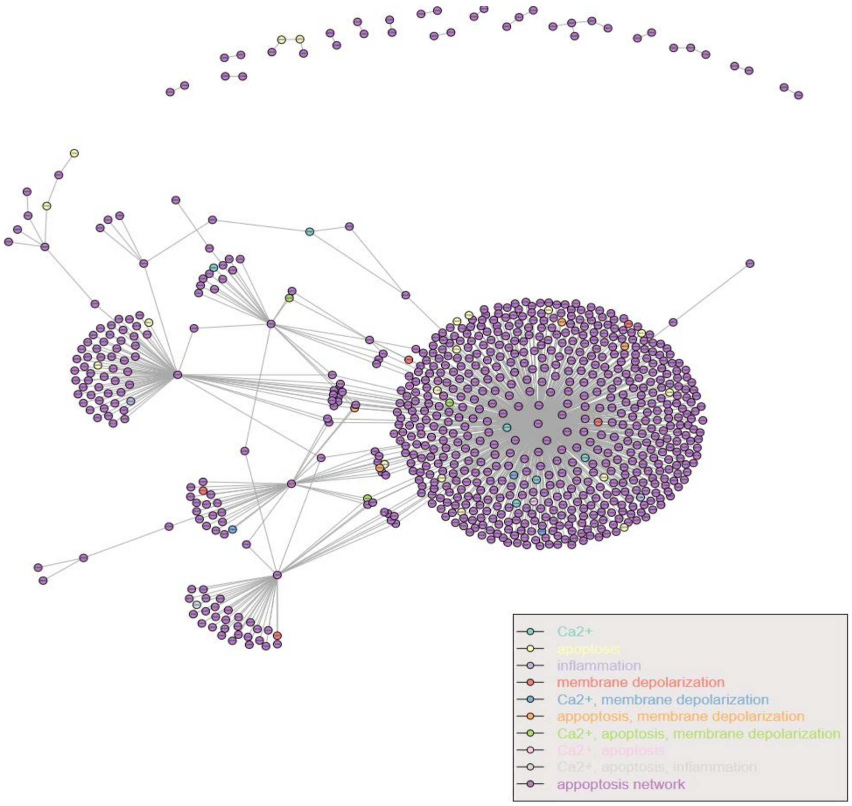

Figure S-5: Network analysis based on Uniprot data base shows our detected proteins (changed expressed proteins in epileptic rats) have important role in calcium homeostasis network.

Figure S-6: original images related to immunohistochemistry to local study of the ADPRC, calreticuline, SNAP 25 and UCH-L1 in hippocampus of control and epileptic rats.

ADPRC	DAPI	Anti-ADPRC	Merge
Control	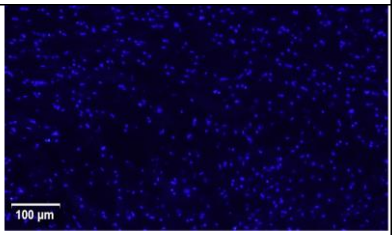	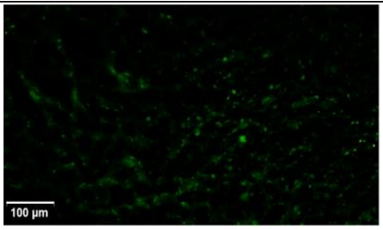	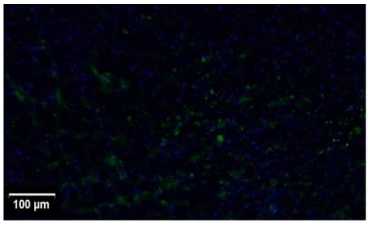
Epilepti<br>c	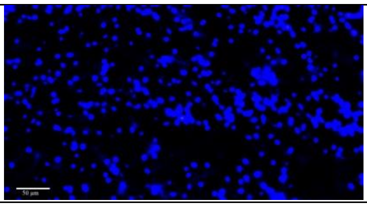	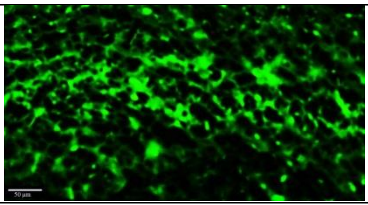	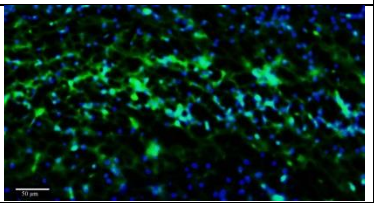

Calreticulin	DAPI	Anti-Calreticulin	Merge
Control	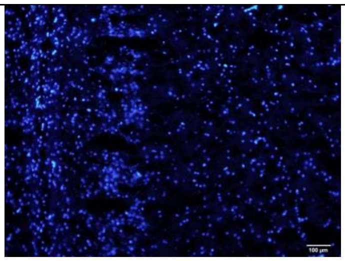	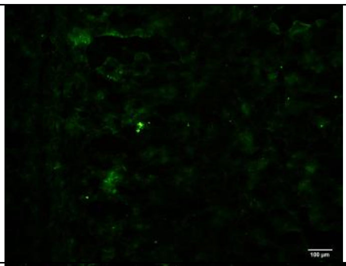	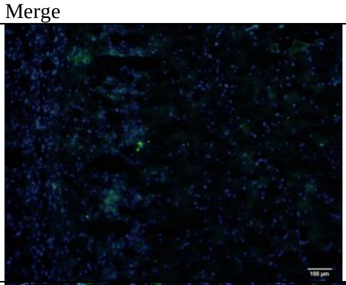
Epileptic	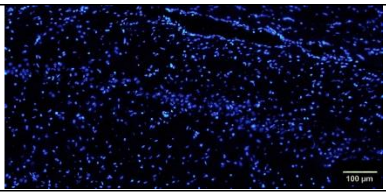	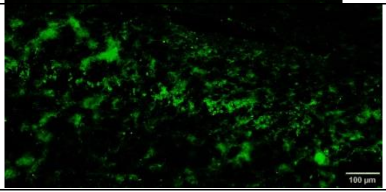	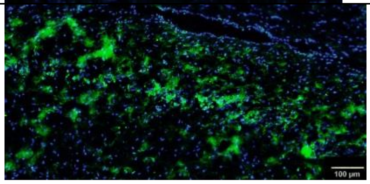

SNAP 25	DAPI	Anti-SNAP 25	Merge
Control	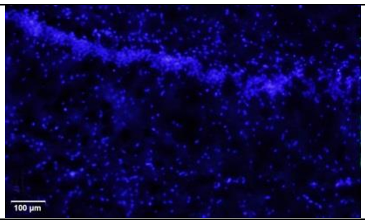	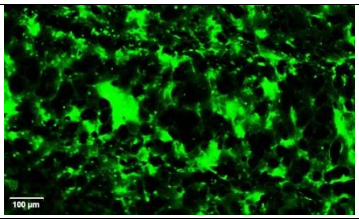	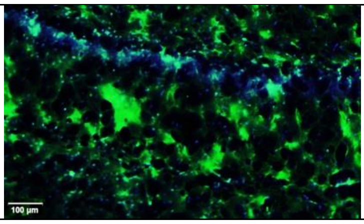

Epileptic	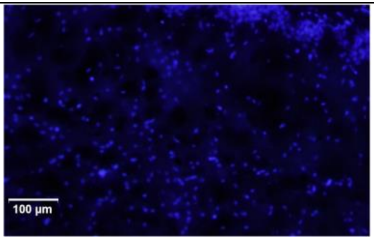	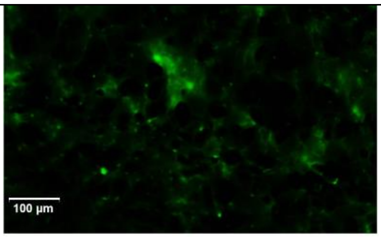	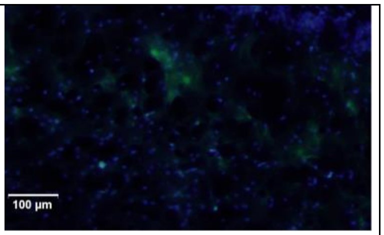

UCH-L1	DAPI	Anti-PGP 9.5	Merge
Control	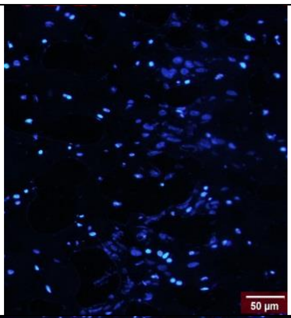	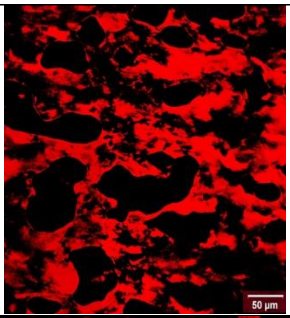	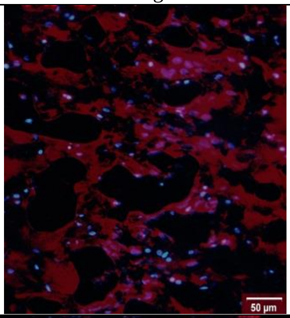
Epileptic	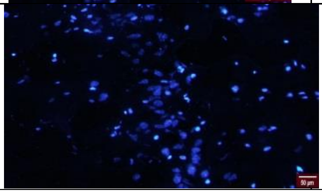	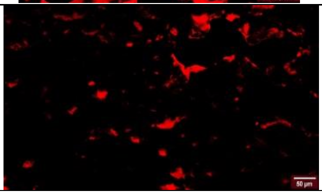	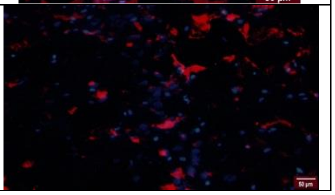

Figure S-7: Original images related to 2-DE gels in experimental rats (epileptic and control).

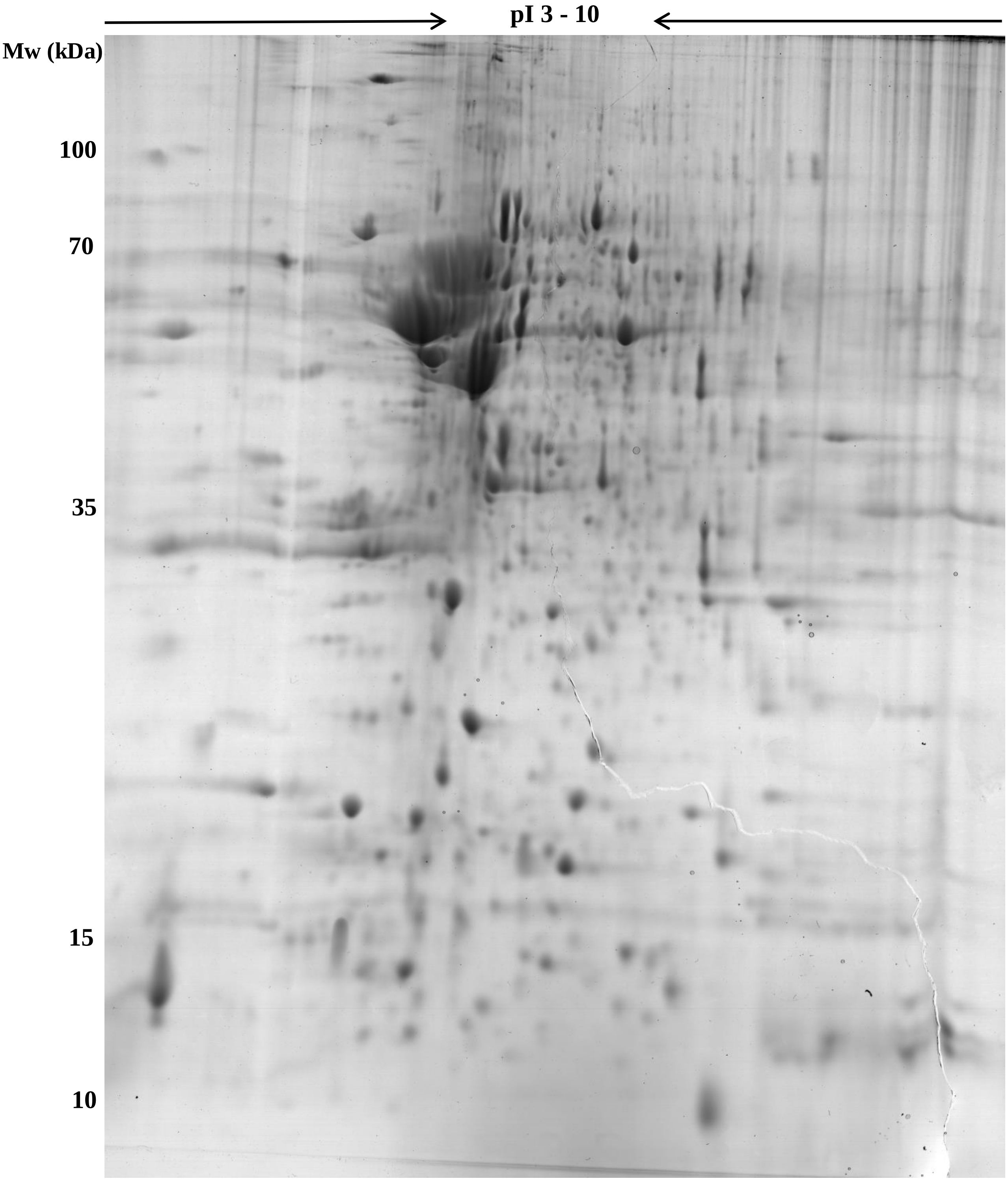

A1: 2-DE gel related to left hippocampus from control rats.

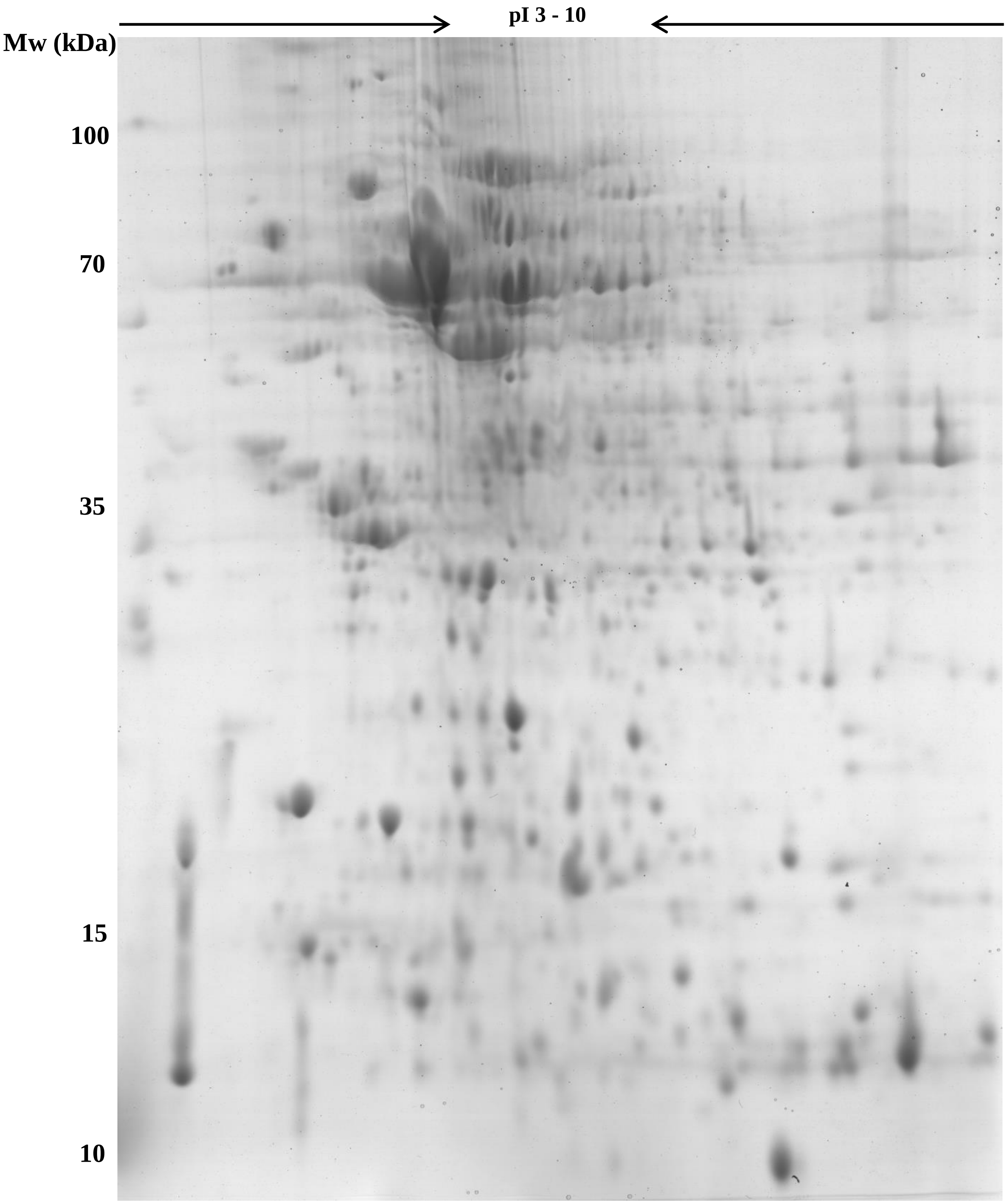

A2: 2-DE gel related to right hippocampus from control rats.

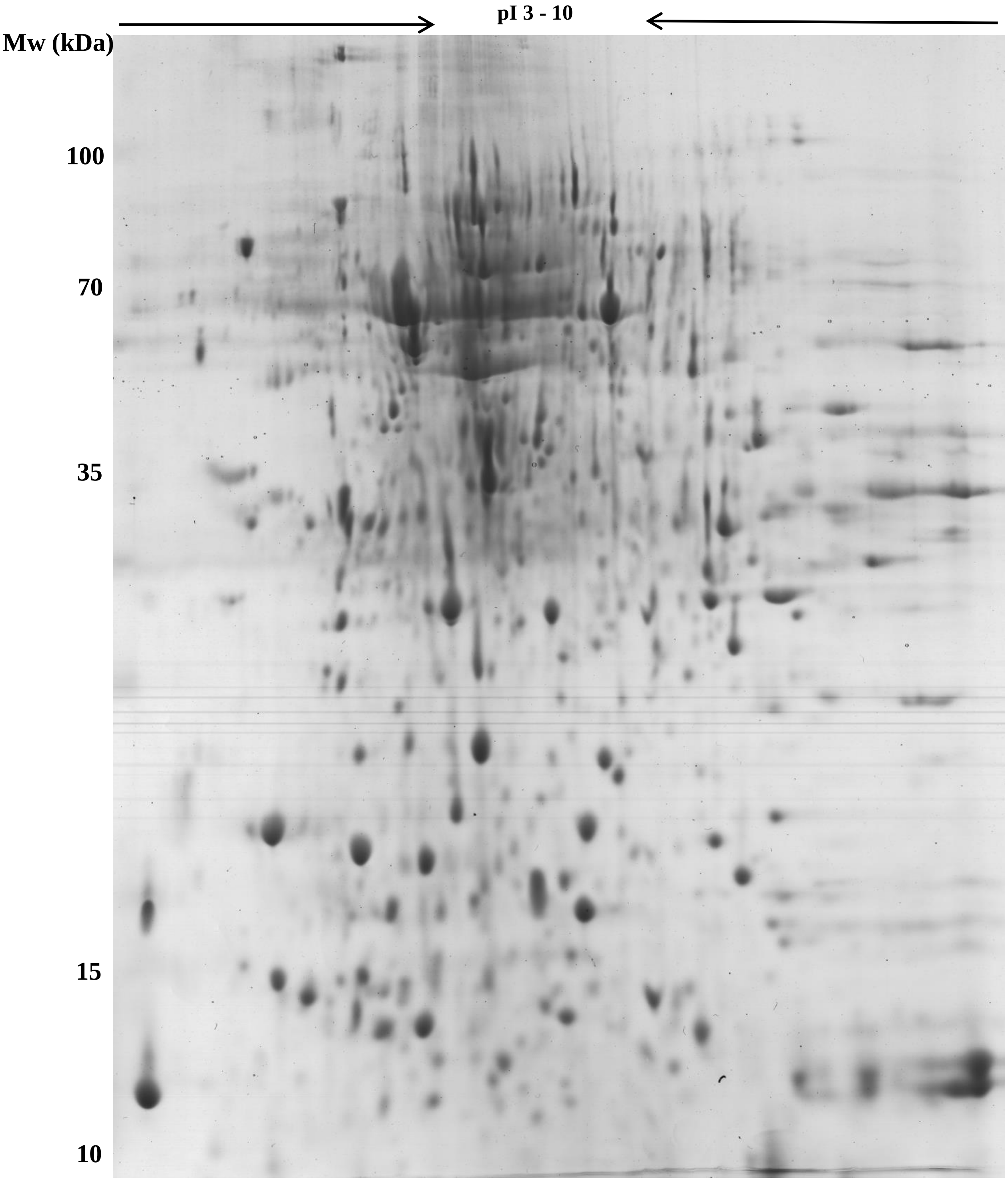

B1: 2-DE gel related to left hippocampus from pilocarpine model of epilepsy.

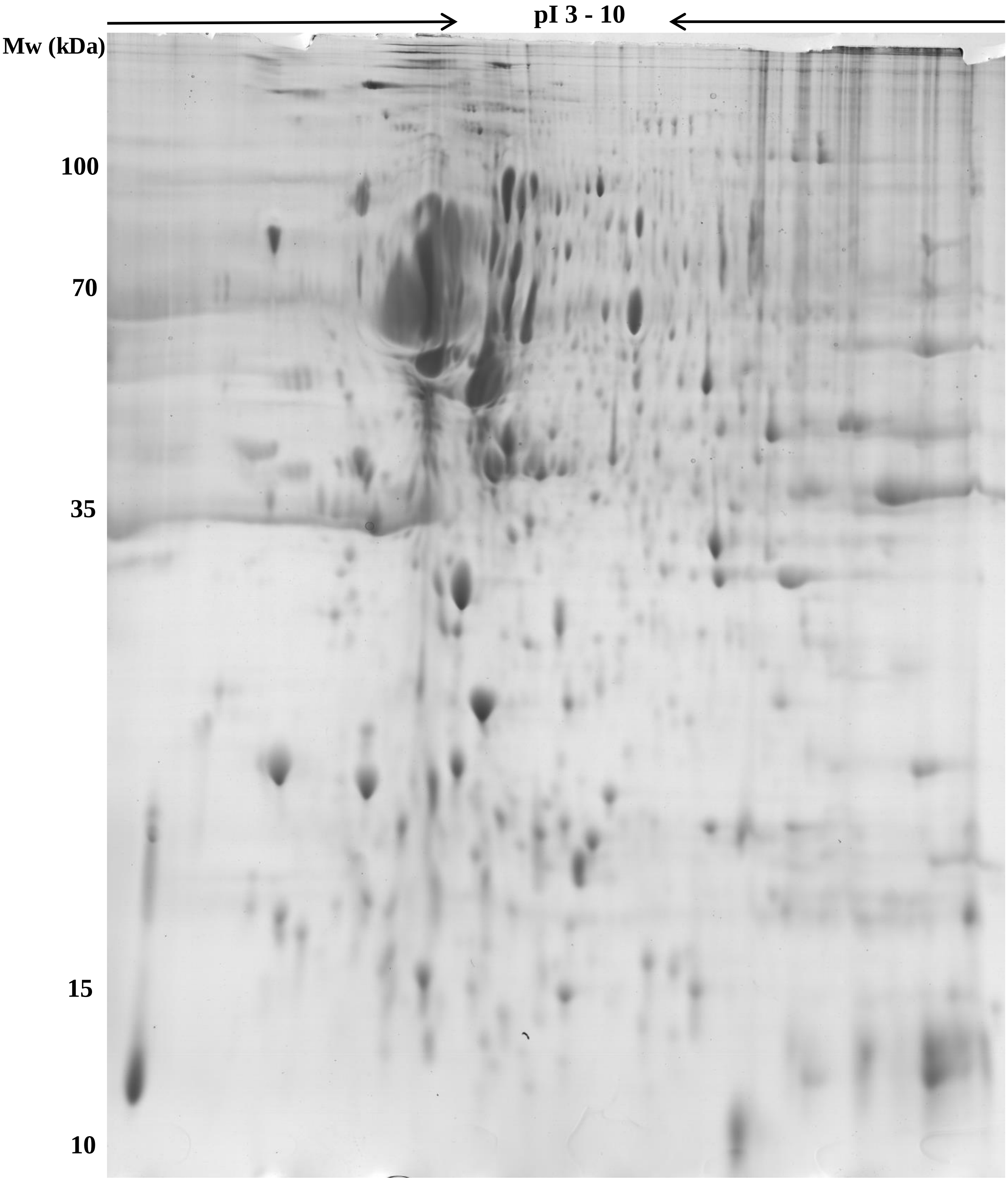

B2: 2-DE gel related to right hippocampus from pilocarpine model of epilepsy.

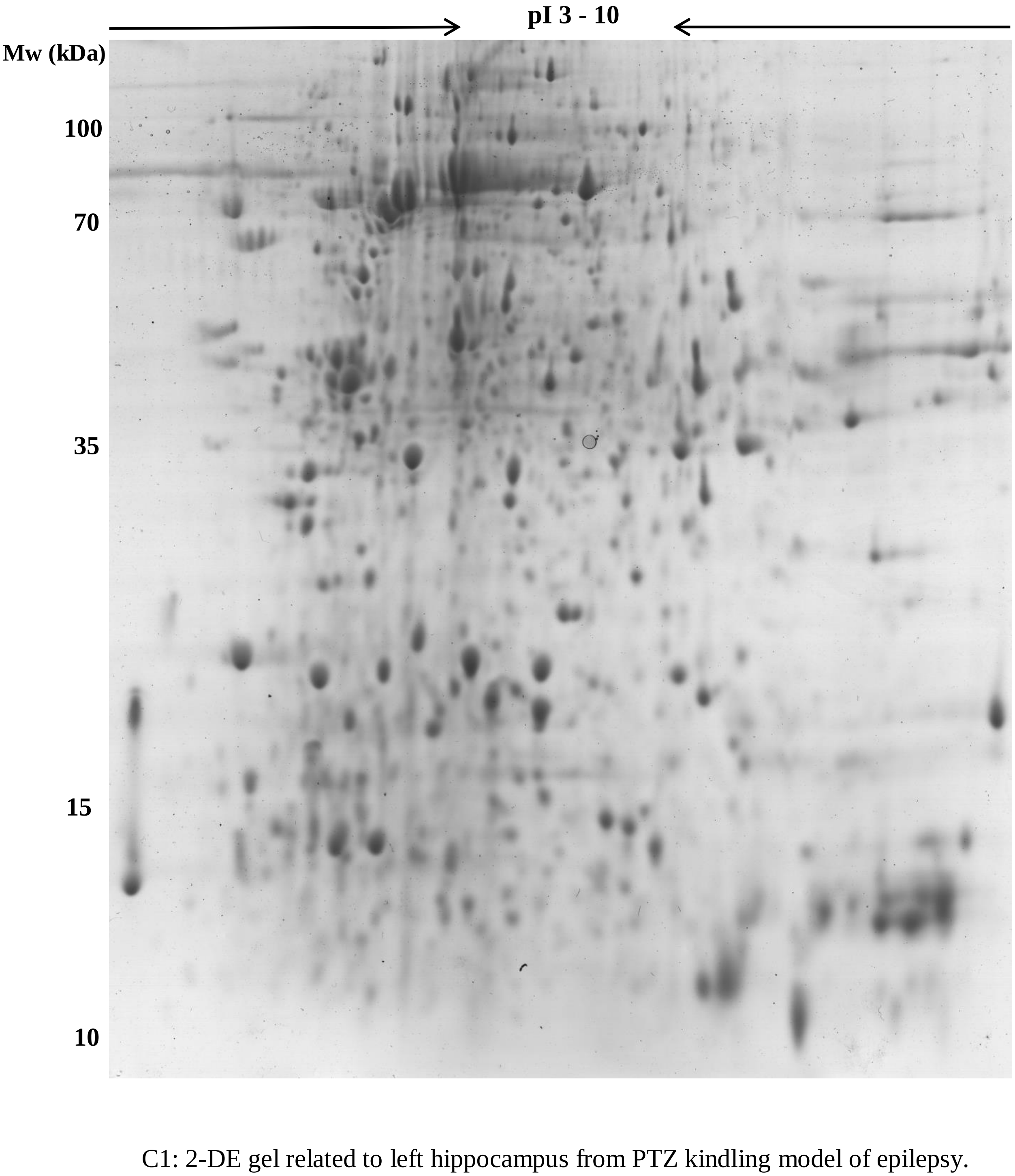

C1: 2-DE gel related to left hippocampus from PTZ kindling model of epilepsy.

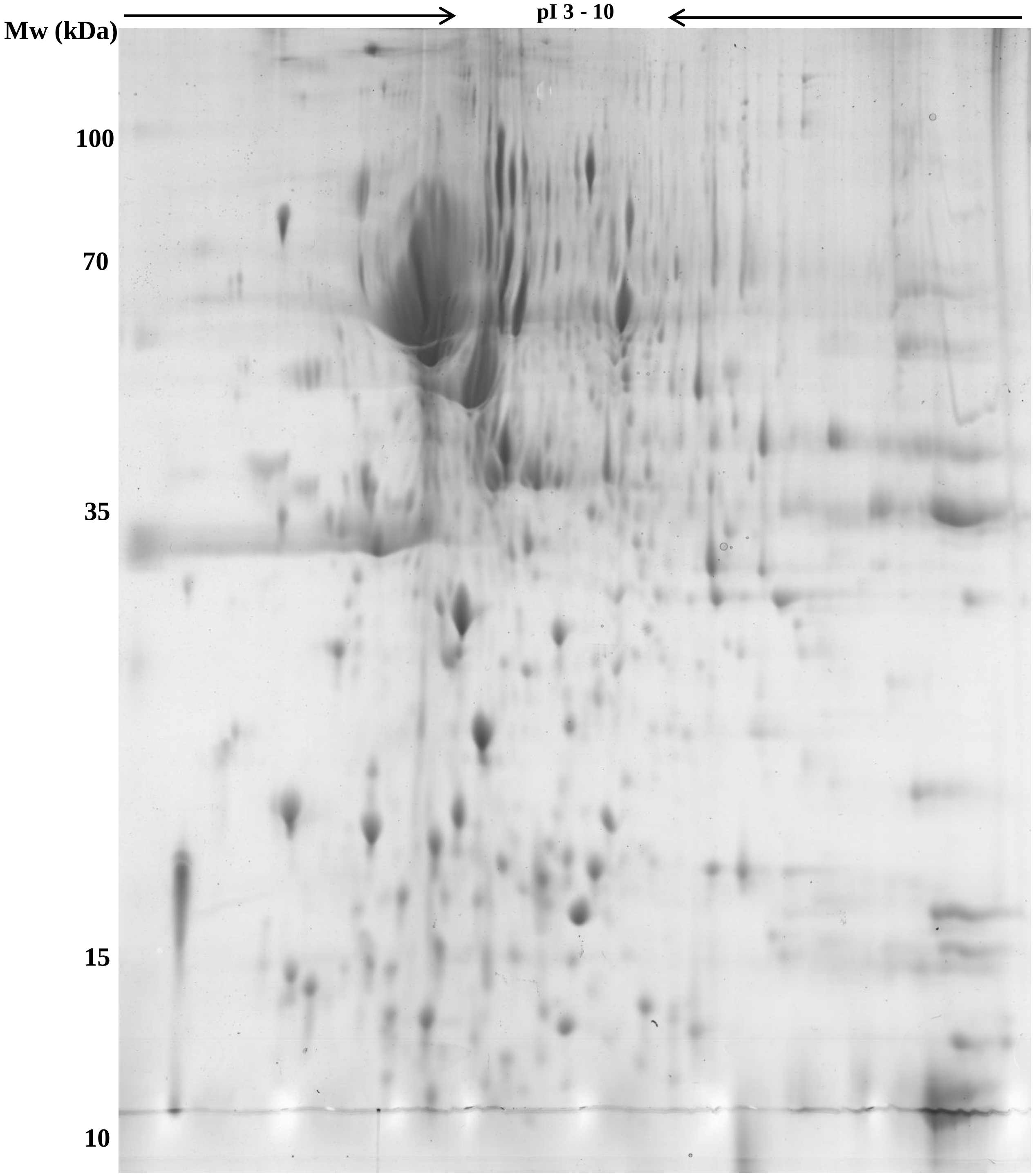

C2: 2-DE gel related to right hippocampus from PTZ kindling model of epilepsy.

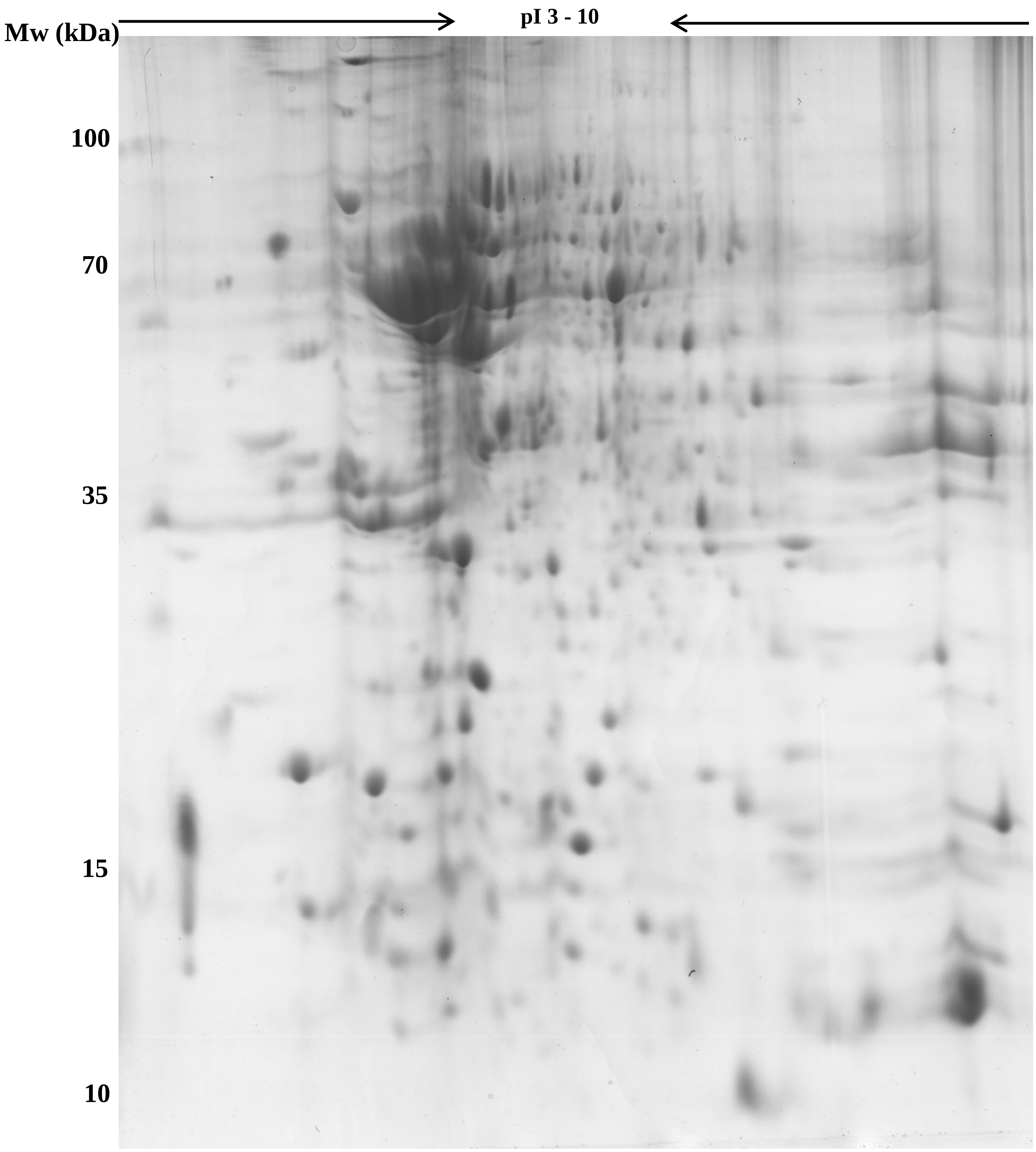

D1: 2-DE gel related to left hippocampus from electrical kindling model of epilepsy.

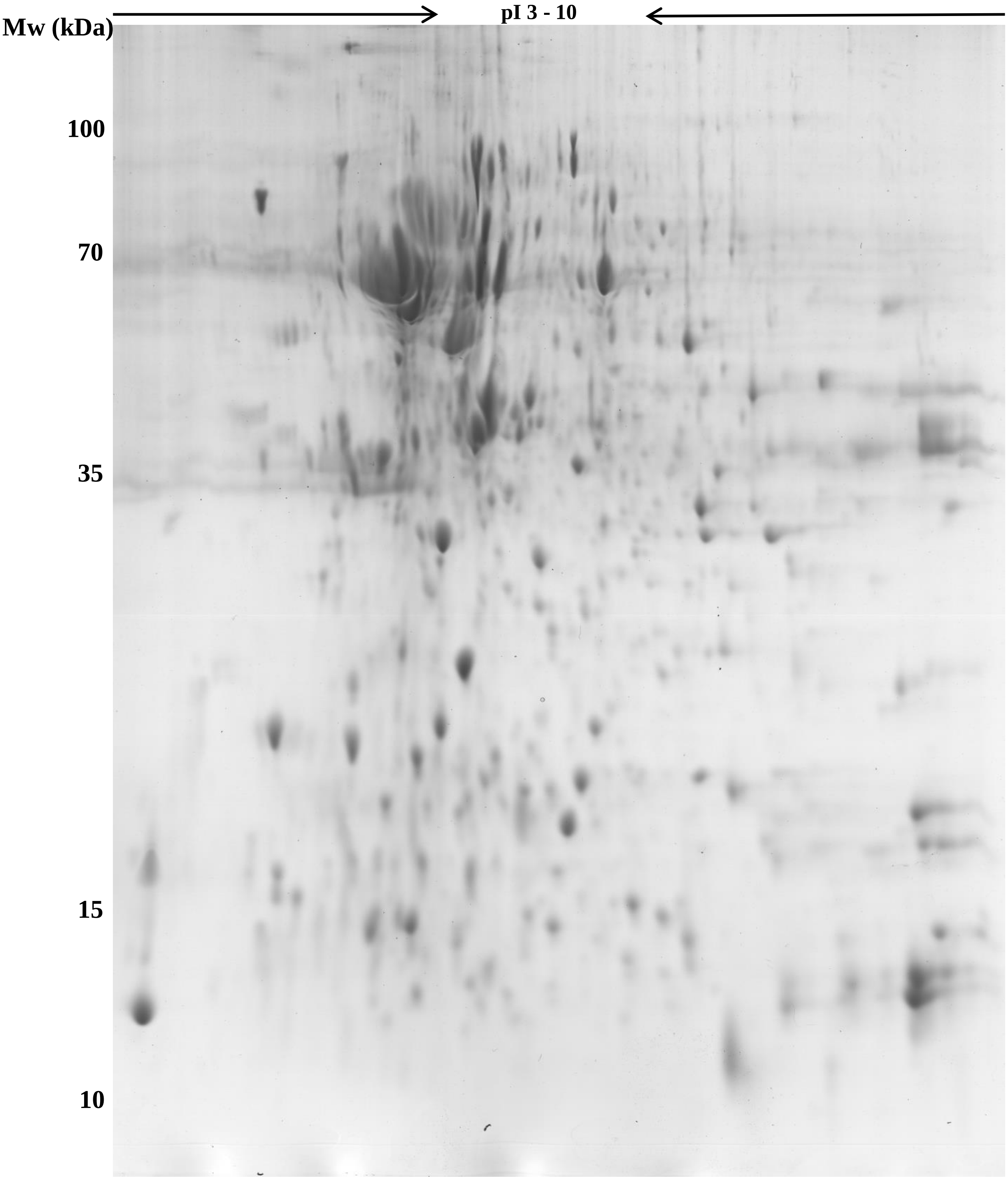

D2: 2-DE gel related to right hippocampus from electrical kindling model of epilepsy.
